# Contrasting genetic predisposition and diagnosis in psychiatric disorders: A multi-omic single-nucleus analysis of the human OFC

**DOI:** 10.1126/sciadv.adq2290

**Published:** 2025-03-07

**Authors:** Nathalie Gerstner, Anna S. Fröhlich, Natalie Matosin, Miriam Gagliardi, Cristiana Cruceanu, Maik Ködel, Monika Rex-Haffner, Xinming Tu, Sara Mostafavi, Michael J. Ziller, Elisabeth B. Binder, Janine Knauer-Arloth

**Affiliations:** ^1^ Department Genes and Environment, Max Planck Institute of Psychiatry, Munich, Germany.; ^2^ International Max Planck Research School for Translational Psychiatry, Munich, Germany.; ^3^ Institute of Computational Biology, Helmholtz Zentrum München, Neuherberg, Germany.; ^4^ School of Medical Sciences, Faculty of Medicine and Health, University of Sydney, Sydney, Australia.; ^5^ Department of Psychiatry, University of Münster, Münster, Germany.; ^6^ Department of Physiology and Pharmacology, Karolinska Institutet, Stockholm, Sweden.; ^7^ Paul G. Allen School of Computer Science and Engineering, University of Washington, Seattle, WA, USA.; ^8^ Department of Psychiatry and Behavioral Sciences, Emory University School of Medicine, Atlanta, GA, USA.

## Abstract

Psychiatric disorders like schizophrenia, bipolar disorder, and major depressive disorder exhibit substantial genetic and clinical overlap. However, their molecular architecture remains elusive due to their polygenic nature and complex brain cell interactions. We integrated clinical data with genetic susceptibility to investigate gene expression and chromatin accessibility in the orbitofrontal cortex of 92 postmortem human brain samples at the single-nucleus (sn) level. Using snRNA-seq and snATAC-seq, we analyzed ~800,000 and 400,000 nuclei, respectively. We observed cell-type–specific dysregulation related to clinical diagnosis and genetic risk. Dysregulation in gene expression and chromatin accessibility associated with diagnosis was pronounced in excitatory neurons. Conversely, genetic risk predominantly affected glial and endothelial cells. Notably, *INO80E* and *HCN2* genes exhibited dysregulation in excitatory neurons’ superficial layers 2/3 influenced by schizophrenia polygenic risk. This study unveils the complex genetic and epigenetic landscape of psychiatric disorders, emphasizing the importance of cell-type–specific analyses in understanding their pathogenesis and contrasting genetic predisposition with clinical diagnosis.

## Teaser

Genetic risk and diagnosis for psychiatric disorders influence gene expression and chromatin accessibility in brain cells.

## INTRODUCTION

Psychiatric disorders, including major depressive disorder (MDD), bipolar disorder, and schizophrenia, have a strong impact on an individual’s quality of life and pose a substantial economic burden, and their most devastating outcome is suicide (*1*). These disorders not only display overlapping symptoms (*2*) but also share a common genetic architecture (*3*, *4*). Genetic correlation analyses have unveiled distinct interconnected clusters among these disorders, indicating their interconnected nature and underscoring the genetic overlap between mood and psychotic disorders (*4*). This shared genetic architecture has been the focus of extensive research (*4*–*6*).

Genome-wide association studies (GWASs) have advanced our understanding of the genetic architecture of psychiatric disorders, uncovering numerous significant genetic variants (*4*, *7*–*9*). Polygenic risk scores (PRSs) have emerged as a pivotal tool for capturing the cumulative genetic risk for a particular trait (*10*), emphasizing the multigenic nature of the etiology of psychiatric disorders. The application of PRS has facilitated a deeper understanding of the relationship between genetic risk and various genomic layers (*11*), such as gene expression and chromatin accessibility, to understand the full spectrum of psychiatric disorders.

In this context, the role of gene expression studies is particularly relevant. Although previous research has identified numerous genes associated with disorders such as schizophrenia, the direction of effects and overlap with GWAS findings often vary, indicating a complex relationship between gene expression changes and genetic susceptibility (*12*). Transcriptome-wide association studies (TWASs), expression quantitative trait loci (eQTLs), and eQTScore (association analyses between PRS and gene expression) analyses have further bridged the gap between GWAS findings and gene expression data (*5*, *12*–*14*). These integrative approaches provide insights into how the identified GWAS variants can influence gene expression, thereby contributing to the pathophysiology and a more nuanced understanding of psychiatric disorders (*15*).

Given that most GWAS variants associated with psychiatric disorders are located in noncoding regulatory elements (*4*, *16*), there is an increased focus on epigenetic studies, which can provide context regarding the intricate relationship between genetics, gene regulation, and environmental factors. In this regard, Bryois *et al.* (*16*) investigated the link between schizophrenia and chromatin accessibility in the prefrontal cortex, identifying their assay for transposase-accessible chromatin using sequencing (ATAC-seq) data as strongly associated with common GWAS variants for schizophrenia. Additionally, Hauberg *et al.* (*17*) observed considerable variability in chromatin accessibility across cell types in different cortical regions, revealing that such diversity may obscure cell-type–specific effects in aggregate studies, thereby underscoring the intricate complexities of epigenetic regulation.

The etiology of psychiatric disorders is notably complex, involving diverse molecular, cellular, and structural alterations across various regions of the human brain, such as the prefrontal cortex, which plays a crucial role in higher cognitive functions and has been linked to various psychiatric conditions (*5*, *7*–*9*, *12*, *18*–*20*). Structural and functional abnormalities in areas like the orbitofrontal cortex (OFC), a key component of the ventral prefrontal cortex, have been widely reported in many psychiatric disorders (*21*). Brodmann area 11 (BA11), a subregion of the OFC, has shown reduced gray matter volume in patients with schizophrenia (*22*) and dysregulation of gene expression and DNA methylation in depressed and suicidal patients (*23*).

The emergence of single-cell sequencing technologies in recent years has revolutionized our ability to conduct high-resolution studies of various tissues at the level of individual cell types (*24*, *25*). These technologies have enabled the creation of single-cell transcriptomic and epigenomic atlases of the human brain, uncovering hundreds of distinct cell types and even thousands of cellular subtypes within millions of cells across different brain regions (*26*, *27*). Such advancements have greatly enhanced our understanding of the cellular specificity of psychiatric disorders, moving from bulk analyses to the more granular single-cell resolution (*19*, *20*, *28*).

Our study aims to explore the molecular landscape of the OFC in psychiatric disorders, using postmortem samples from patients and controls. By examining differential gene expression and chromatin accessibility at the level of single cells, we uncovered key pathways and functions altered across various cortical cell types and how these changes relate to both genetic predisposition and clinical diagnosis. These findings provide insights into the molecular underpinnings of psychiatric disorders in specific cortical cell types, illustrating how genetic risk factors translate into clinical symptoms and may inform more targeted diagnostics and therapeutic strategies.

## RESULTS

### Single-nucleus multi-omic profiling identifies distinct cell types in the human OFC

**Fig. 1.**
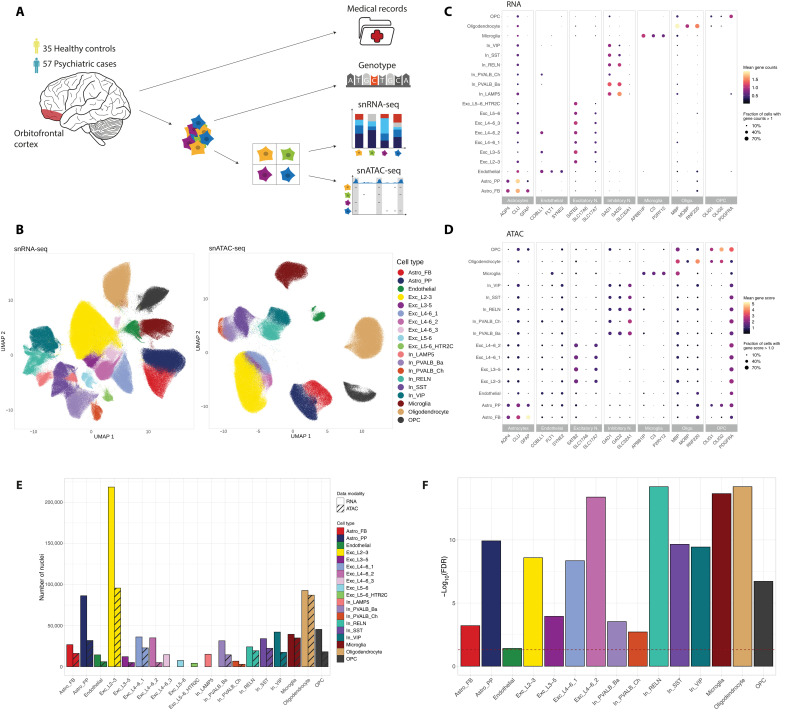
Single-nucleus transcriptomic and epigenomic profiling in the OFC. (**A**) Schematic representation of experimental procedures and data modalities. Nuclei were extracted from the OFC of 57 cases and 35 controls. Single-nucleus RNA-seq (snRNA-seq) and single-nucleus ATAC-seq (snATAC-seq) data were integrated with genotype data and medical records. (**B**) Uniform manifold approximation and projection (UMAP) representations of snRNA-seq (~800,000 nuclei) and snATAC-seq (~400,000 nuclei) data colored by the assigned cell-type labels. Nineteen cell types were assigned to the snRNA-seq data and 15 to the snATAC-seq data. (**C**) Dot plot showing the gene counts in snRNA-seq data of representative marker genes, grouped by major cell types. Color indicates the mean gene counts and size of dots represents the fraction of nuclei with a gene count > 1. (**D**) Dot plot showing the gene score levels in snATAC-seq data of representative marker genes, grouped by major cell types. Color indicates the mean gene score level and size of dots represents the fraction of nuclei with a gene score > 1.0. (**E**) Number of nuclei obtained per cell type following QC colored by cell type. Data modality is indicated by hatching. (**F**) Significance of differences in cell-type proportions between snRNA-seq and snATAC-seq data. Height of the bar represents −log_10_-transformed false discovery rate (FDR) values of a two-sided Wilcoxon signed-rank test, and the dashed red line corresponds to the FDR cutoff of 0.05.

To unravel cell-type–specific molecular alterations in psychiatric disorders within the OFC, we analyzed postmortem brain samples (BA11) using single-nucleus RNA sequencing (snRNA-seq) and single-nucleus ATAC-seq (snATAC-seq), complemented by genotype information and demographic and clinical variables (Fig. 1A). Our cohort was composed of 92 donors, including 35 controls and 57 cases (*n*_schizophrenia_ = 38, *n*_schizoaffective_ = 7, *n*_MDD_ = 7, and *n*_bipolar_ = 5). Case and control groups were matched for sex, age, postmortem interval (PMI), and brain pH; see table S1.

Following stringent quality control (QC) measures, we obtained high-quality transcriptomic data from 787,046 nuclei, averaging 9046 nuclei per donor (range, 3895 to 15,693; table S2). Each nucleus had a median of 3887 unique molecular identifiers (UMIs), detecting a median of 2205 genes. Additionally, chromatin accessibility data were acquired for 399,439 nuclei, averaging 4438 nuclei per donor (range, 982 to 8707; table S2) with a median of 7071 ATAC-seq fragments per nucleus. snRNA-seq and snATAC-seq data enabled the comprehensive profiling of all major cortical cell types, including excitatory and inhibitory neurons across different cortical layers, endothelial cells, and glial subtypes, like astrocytes, microglia, oligodendrocytes, and oligodendrocyte precursor cells (OPCs). We successfully identified 19 distinct cell types in snRNA-seq data, with 15 of these also present in the snATAC-seq data (Fig. 1, B to E). This identification aligns well with the expected diversity of cell types in the human brain and demonstrates the robustness of our method. While the number of nuclei per cell type exhibited heterogeneity, both among different cell types and between snRNA-seq and snATAC-seq datasets (Fig. 1E), a high median Pearson correlation coefficient of 0.86 was observed between the cell-type proportions of RNA-seq and ATAC-seq data across donors (fig. S1A). Cell-type proportions differed significantly [false discovery rate (FDR) ≤ 0.05] between the RNA-seq and ATAC-seq modalities for all cell types (Fig. 1F and table S3). Conversely and in agreement with previous research findings (*20*), no significant difference in cell-type proportions between cases and controls within each data modality was observed (fig. S1, B and C).

### Cell-type–specific alterations in psychiatric disorders: Distinct patterns in differential gene expression and chromatin accessibility

**Fig. 2.**
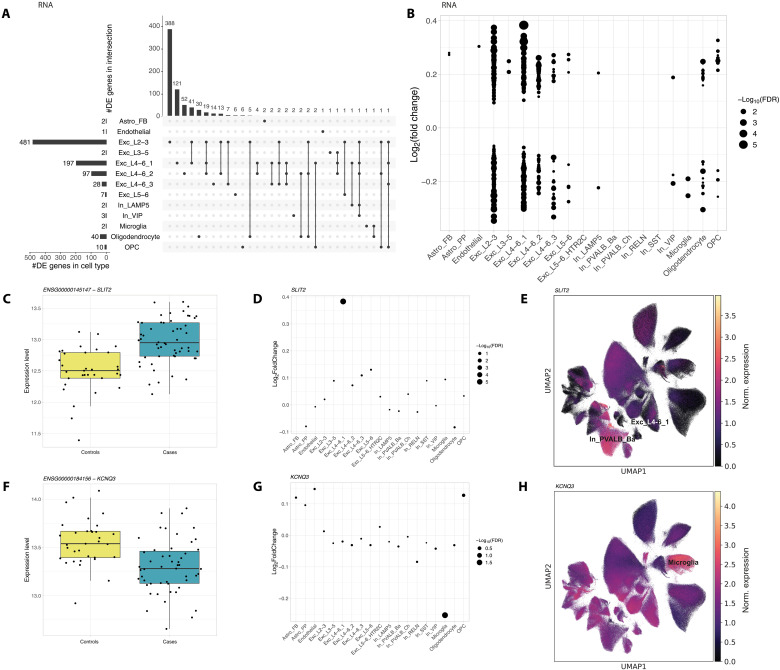
Transcriptional alterations between psychiatric cases and controls. (**A**) UpSet plot showing the number of differentially expressed (DE) genes (FDR ≤ 0.1) per cell type (left) and the overlap of DE genes between cell types (right). (**B**) Dot plot of DE genes with log_2_ fold change (FC) on the *y* axis and −log_10_-transformed FDR values represented by dot size. (**C** and **F**) Box plot of normalized gene expression level for *SLIT2* (C) and *KCNQ3* (F) in controls and cases. (**D** and **G**) Dot plot of log_2_ FCs of *SLIT2* (D) and *KCNQ3* (G) in each cell type. Dot size represents −log_10_-transformed FDR values. (**E** and **H**) UMAP representation of snRNA-seq data colored by normalized expression of *SLIT2* (E) and *KCNQ3* (H).

As there were no significant differences in cell-type proportions between cases and controls within each data modality, we moved our focus on more detailed molecular analyses. To investigate transcriptional alterations associated with psychiatric disorders, we conducted differential expression analyses, contrasting cases (*n* = 57) and controls (*n* = 35) within each cell type (*n* = 19). Given that most cases in our cohort were schizophrenia cases, we also performed an additional analysis focusing solely on the schizophrenia cohort, which is detailed in the Supplementary Materials. The extent of significantly differentially expressed (DE; FDR ≤ 0.1) genes varied greatly across cell types, ranging from 0 to 481 (Fig. 2A and table S7). Notably, a high abundance of DE genes was observed within multiple subtypes of excitatory neurons, which also exhibited the most pronounced log_2_-transformed fold changes (FC = [−0.35, 0.38]; see Fig. 2B). More than 50% of DE hits were uniquely dysregulated in one cell type only (Fig. 2A), highlighting the distinct transcriptional signatures across cell types. However, the cell types displaying the greatest number of DE genes also have the highest nucleus count and the largest number of genes evaluated for DE, suggesting a proportional relationship between these variables (see fig. S3, B and C).

Notably, *Slit Guidance Ligand 2* (*SLIT2*) on chromosome 4 and *Potassium Voltage-Gated Channel Subfamily Q Member 3* (*KCNQ3*) on chromosome 8 demonstrated unique regulatory patterns (Fig. 2, C to H). *SLIT2* displayed the highest up-regulation (FC = 0.38 and lowest FDR = 1.38 × 10^−6^) in excitatory neurons of layers 4 to 6, cluster 1 (Exc_L4-6_1; Fig. 2, C and D), despite not exhibiting the highest expression in this cell type [mean exp. = 0.32, compared to 1.66 in basket cells (In_PVALB_Ba); Fig. 2E]. While *SLIT2* is known for its role in axon guidance and has been implicated in depression and anxiety-like behaviors in mice (*29*), which are symptoms shared across various psychiatric disorders, our results indicate a cell-type–specific dysregulation in the human cortex. *KCNQ3* was uniquely down-regulated in microglia (FC = −0.25 and FDR = 0.02), a contrast to its FCs in other cell types (FCs > −0.05; Fig. 2, F and G). Exhibiting the highest expression in microglia (mean exp. = 1.75; Fig. 2H) and previously linked to bipolar disorder (*30*), the specific down-regulation of *KCNQ3* in microglia offers insights into the cellular mechanisms that may contribute to a range of psychiatric disorders, particularly in the context of neuroinflammation and microglial function.

To evaluate how both the cross-disorder and the schizophrenia-specific results of our study align with previous studies, we correlated our effect sizes with those reported in a single-cell RNA-seq (scRNA-seq) study of schizophrenia in the prefrontal cortex by Ruzicka *et al.* (*20*); see Materials and Methods. Among the various correlations observed between the effect sizes of the two studies, those between corresponding cell types were notably the highest for the cross-disorder, as well as the schizophrenia-specific results (fig. S4, A and B), indicating a broad consistency with previous findings.

From the DE genes, we identified within individual cell types (*n* = 872), only 44% (*n* = 387) exhibited a significant difference in gene expression on the full pseudobulk level, which is the aggregated signal of all cell types. Of all DE genes identified from the full pseudobulk data (*n* = 511), 57% (*n* = 291) were significant in at least one individual cell type (fig. S4, C and D), which highlights the importance of studying the single-cell level.

**Fig. 3.**
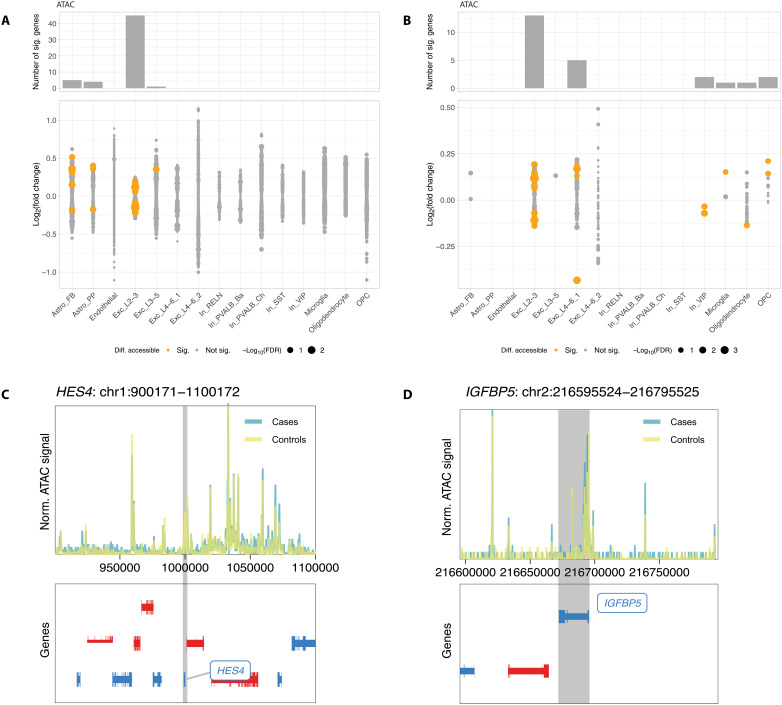
Epigenomic alterations between psychiatric cases and controls. (**A** and **B**) Results of differential chromatin accessibility (DA) analysis when testing all genes passing filtering step (A) and only DE genes (B). Bar plot on top shows the number of significant DA genes per cell type (FDR ≤ 0.1). Log_2_ FCs for all tested genes are shown in dot plot with color indicating the DA significance and dot size indicating the −log_10_-transformed FDR value. No overlap was observed between DA genes in different cell types. (**C** and **D**) Genome tracks visualizing normalized ATAC signal in a 100-kb window surrounding the gene body of *HES4* (C) and *IGFBP5* (D). *HES4* in excitatory neuron layers 4 to 6, cluster 1 (Exc_L4-6_1), had FDR values of 0.04 (RNA) and 2.21 × 10^−3^(ATAC) with FCs of −0.28 (RNA) and −0.43 (ATAC). Similarly, *IGFBP5* in OPCs showed FDR values of 1.70 × 10^−5^ (RNA) and 0.08 (ATAC) with FCs of 0.33 (RNA) and 0.21 (ATAC).

To complement findings from our DE analysis, we examined variations in chromatin accessibility between cases and controls across shared 15 cell types. Our focus was on differences in gene scores, a quantitative measure of gene activity influenced by accessible chromatin. Only a small number of significant accessibility alterations (DA; FDR ≤ 0.1) were found in two clusters of excitatory neurons [excitatory neurons’ layers 2/3 (Exc_L2-3), *n* = 45, and excitatory neurons’ layers 3 to 5 (Exc_L3-5), *n* = 1] and in astrocytes [fibrous (Astro_FB), *n* = 5, and protoplasmic (Astro_PP), *n* = 4]; see Fig. 3A and table S8. Only five of the DA genes overlapped with DE genes previously identified in the same cell type. When restricting the DA analysis to DE genes within the respective cell type, we found a subset also demonstrating significant alterations in chromatin accessibility. The maximum number of DE/DA genes was 13 in excitatory neurons in superficial layers 2/3 (Fig. 3B and table S9). Notably, discrepancies in regulation direction between transcriptomic and epigenomic data were noted in 8% of DE/DA genes (2 of the 24 genes; fig. S5A). Among the 22 genes with congruent regulatory patterns in both datasets, not only *Hes family BHLH transcription factor 4* (*HES4*) in excitatory neuron layers 4 to 6, cluster 1 (Exc_L4-6_1), and *insulin-like growth factor-binding protein 5* (*IGFBP5*) in OPCs exhibit the most pronounced FCs (Fig. 3, C and D), but also *SLIT2* in excitatory neurons’ layers 4 to 6, cluster 1 (Exc_L4-6_1), is a DE/DA gene. These findings, including the discrepancies, imply a complex regulatory landscape and suggest that additional regulatory mechanisms may influence the gene expression.

### Differential transcriptomic and epigenomic patterns in high– versus low–genetic risk donors highlight chromatin accessibility variations

**Fig. 4.**
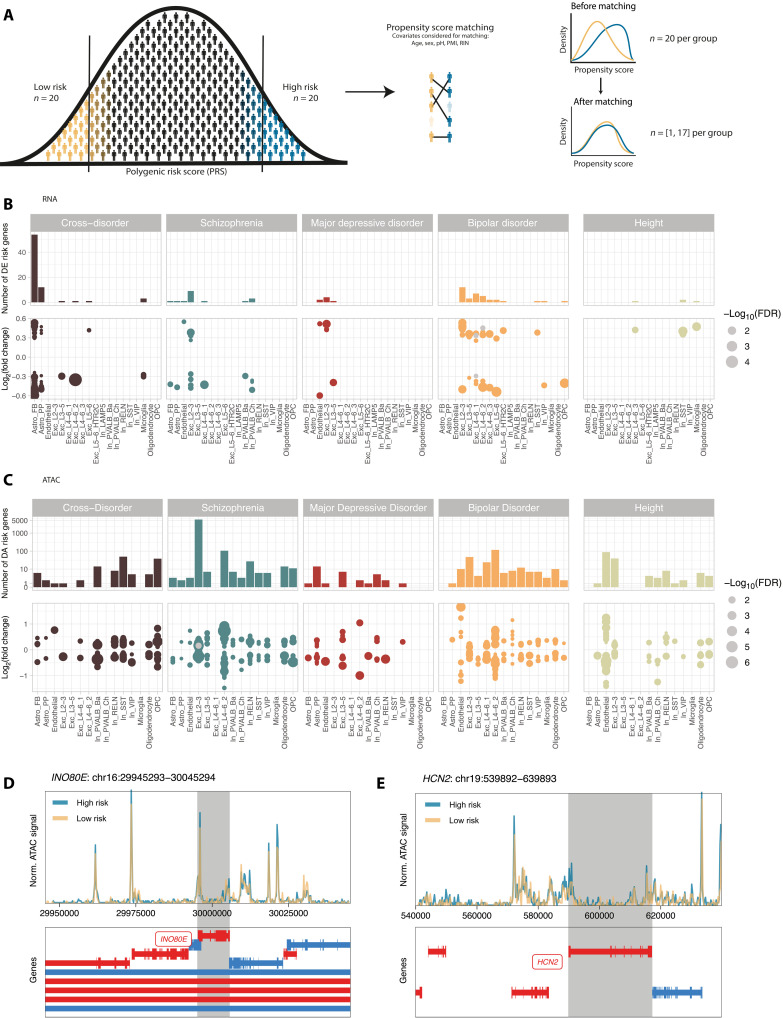
Gene regulatory differences between extreme–genetic risk groups. (**A**) Schematic overview of the definition of extreme–genetic risk groups using propensity score matching (see Materials and Methods). (**B** and **C**) Combined bar plots and dot plots visualizing the number, effect sizes, and significance of DE (B) and DA (C) risk genes (FDR ≤ 0.1) between donors at high and low genetic risk quantified by PRS on the basis of five different GWAS. Each dot represents a significant genetic risk gene (FDR ≤ 0.1). Color indicates the GWAS, while dot size represents −log_10_-transformed FDR values. Gray dots in (B) represent DE genetic risk genes that are also DE genes between cases and controls in the same cell type, while gray dots in (C) represent DA genetic risk genes that are also DE risk genes in the same cell type. (**D** and **E**) Genome tracks visualizing normalized ATAC signal in a 100-kb window surrounding the gene body of *INO80E* (D) and *HCN2* (E).

To disentangle the influence of genetic predisposition on gene expression and chromatin accessibility, we used PRSs from psychiatric GWASs, including cross-disorder phenotype (*4*), schizophrenia (*7*), MDD (*8*) and bipolar disorder (*9*), and height (*31*) as a nonpsychiatric trait (table S4). Focusing on the extreme PRS groups, matched for confounding variables (see Materials and Methods, figs. S6 and S7, and table S5), we found significant DE risk genes in 3 to 10 of the 19 cell types for each GWAS trait (Fig. 4B and table S10). Fifty-four DE risk genes were found in the fibrous astrocytes (Astro_FB) for the cross-disorder phenotype and scattered hits across other cell types (*n* = 18 hits in five cell types). Bipolar disorder DE risk genes were detected primarily in excitatory neurons (*n* = 32 of 35 hits) overlapping partially with the DE genes between cases and controls (*n* = 3 of 35 hits; Fig. 4B, gray dots). Genetic risk for schizophrenia was associated with changes in multiple cell types (*n* = 17 hits in seven cell types), while fewer MDD risk genes emerged as significant (*n* = 7 hits in three cell types). DE risk genes exhibited larger effect sizes than DE genes for clinical diagnoses (median absolute FC_PRS_ = [0.29, 0.55] versus median absolute FC_diagnosis_ = [0.18, 0.30] per cell type; see fig. S10A). Notably, three DE risk genes were identified across three different cell types for height, which are distinct from the DE risk genes for the psychiatric phenotypes.

When investigating DA between extreme PRS groups (DA risk genes), we identified 6418 DA risk genes across cell types and phenotypes (Fig. 4C and table S11), contrasting with 141 DE risk genes. These genes were primarily enriched in excitatory neurons’ layers 2/3 (Exc_L2-3, *n* = 5645 DA risk genes). Also, DA risk genes exhibited larger effect sizes than DA genes for clinical diagnoses (median absolute FC_PRS_ = [0.15, 0.74] versus median absolute FC_diagnosis_ = [0.12, 0.35] per cell type; see fig. S10B). Notably, despite identifying DA risk genes for height, only one overlapped with bipolar disorder DA risk genes. The overlap between DA and DE risk genes was minimal with only two genes (Fig. 4C, gray dots), *hyperpolarization-activated cyclic nucleotide-channel 2* (*HCN2*) and *IN080 complex subunit E* (*INO80E*), being both DE (FCs = 0.36 and 0.26 and FDR = 0.06 and 0.09, respectively) and DA (FCs = 0.14 and 0.16 and FDR = 0.05 and 0.03, respectively) for schizophrenia risk in excitatory neurons’ layers 2/3 (Exc_L2-3). Genomic tracks surrounding *HCN2* and *INO80E* illustrate different ATAC coverage for the high– and low–schizophrenia risk groups (Fig. 4, D and E). In bulk GTEX data, *HCN2* is mostly expressed in the heart and the nervous system (fig. S9E) (*32*) and contributes to pacemaker currents (*33*), while *INO80E* is expressed across all tissues (fig. S9E) (*32*). *INO80E* is involved in adenosine 5′-triphosphate–dependent chromatin remodeling, DNA replication, and repair (*34*).

In the analysis of the overlap between diagnosis-related genes and genetic risk genes, our findings revealed a distinct cellular specificity. The molecular response in neurons was influenced by both diagnosis-related genes (DE or DA genes) and genes associated with genetic risk (DE or DA risk genes). In contrast, glial cells predominantly exhibited molecular alterations linked to genetic risk factors. Specifically, 81% of gene alterations in OPCs, 76% in microglia, and more than 90% in both fibrous (Astro_FB) and protoplasmic astrocytes (Astro_PP) were linked to genetic risk rather than disease status. Endothelial cells were also primarily influenced by genetics, with 95% of changes tied to genetic risk (fig. S11).

### Disease-relevant pathway enrichment in microglia is uncovered by transcriptomic profiling

**Fig. 5.**
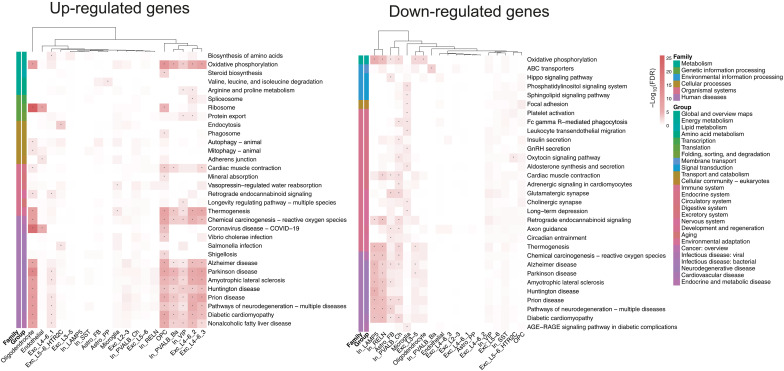
Disease-relevant pathway enrichments. Heatmaps of KEGG pathway enrichment results for the 250 most up- and down-regulated DE genes per cell type comparing cases and controls. All pathways significantly enriched in at least one cell type are included in the heatmap, with color representing −log_10_-transformed FDR values and asterisks indicating significance (FDR ≤ 0.05). Pathway annotations on the left indicate their pathway group and family, and dendrograms visualize *k*-means clustering of cell types according to enrichment results.

To explore the biological processes and functions affected by genes differentially regulated due to diagnosis or genetic predisposition within different cell types, we conducted Kyoto Encyclopedia of Genes and Genomes (KEGG) pathway enrichments. Due to some cell types having very few or no significant hits, we performed these enrichments for each cell type using the 250 most up- and down-regulated DE and DA genes and DE and DA risk genes (see Materials and Methods) to ensure consistent analysis across all cell types. For the top DE genes between cases and controls, down-regulated genes in microglia were distinctively enriched for pathways like long-term depression (FDR = 0.04) and cell-cell interaction mechanisms, such as focal adhesion (FDR = 0.04), setting them apart from other cell types (Fig. 5). Furthermore, top DE genes highlighted distinct pathways in the nervous and endocrine systems (e.g., various synapses or endocannabinoid signaling) enriched for down-regulated genes in fibrous astrocytes (Astro_FB), chandelier cells (In_PVALB_Ch), and microglia. Pathways related to neurodegenerative diseases and oxidative phosphorylation showed significant enrichment in both up- and down-regulated genes in different cell types. Notably, the Ribosome pathway exhibited significant enrichment, particularly in up-regulated genes especially in oligodendrocytes (FDR = 5.18 × 10^−29^), alongside moderate up-regulation observed in OPCs (FDR = 7.20 × 10^−7^) and endothelial cells (FDR = 6.54 × 10^−11^). Many pathways enriched in up- and down-regulated DE risk genes reflecting genetic risk (fig. S8, A to D) overlap with the pathways enriched in genes altered between cases and controls, while the respective cell types exhibiting the enrichment are often different. To further investigate the impact of genetic risk on gene expression, we performed H-MAGMA gene-set analysis (*35*). Detailed results of this analysis can be found in the Supplementary Materials (fig. S8F).

For chromatin accessibility alterations between cases and controls, pathway enrichment analysis revealed no significant enrichments for most cell types (fig. S5B), and, for extreme–genetic risk groups, it revealed only few significant pathways (fig. S9, A to D), which can be attributed to the DA genes’ involvement in separate biological processes rather than shared pathways, and different sizes of background sets.

### Schizophrenia polygenic risk influences *INO80E* and *HCN2* regulation in excitatory neurons in superficial layers 2/3, independent of diagnosis

**Fig. 6.**
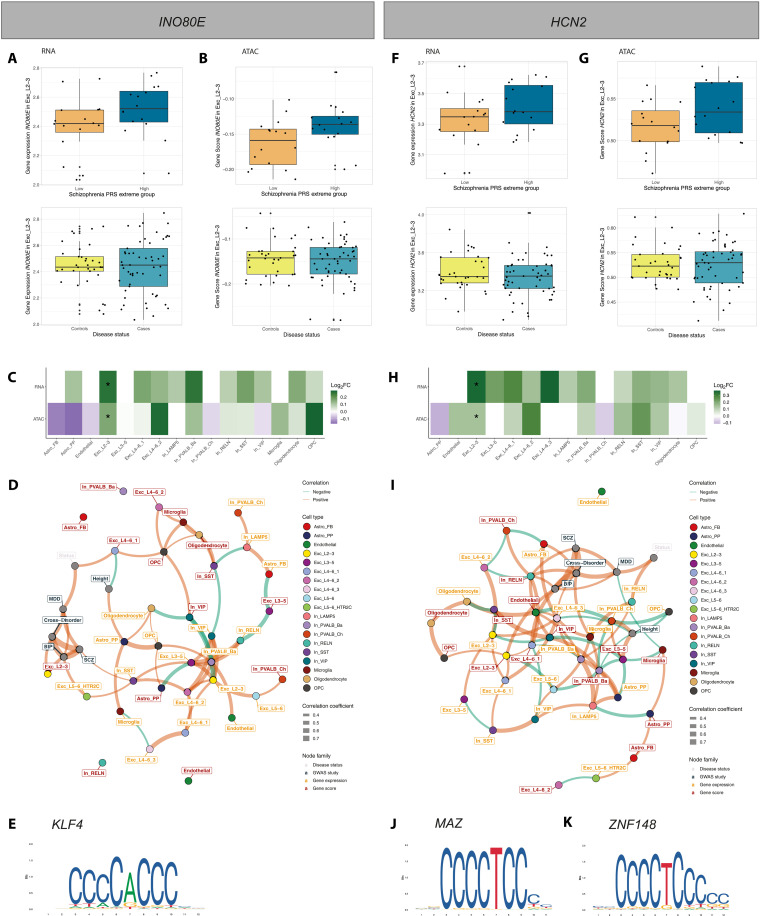
Cell-type–specific gene regulation of *INO80E* and *HCN2* related to genetic risk for schizophrenia. (**A**, **B**, **F**, and **G**) Box plots showing gene expression (A and F) and chromatin accessibility (B and **C**) of *INO80E* (A and B) and *HCN2* (F and G) for extreme–genetic risk groups as well as disease status. (C and **H**) Heatmap visualizing the log_2_ FCs of *INO80E* from DE and DA genetic risk analysis for schizophrenia. Asterisks indicate significance (FDR ≤ 0.1). (**D** and **I**) Correlation-based network for *INO80E* (D) and *HCN2* (I) inferred from PRS for cross-disorder phenotypes, bipolar disorder, MDD, schizophrenia, and height, disease status, as well as gene expression and chromatin accessibility across cell types. All nominally significant correlations are shown (*P* ≤ 0.05). Node color indicates the cell type, color of node labels indicates the node family/data modality, edge thickness relates to correlation strength, and edge color indicates whether the correlation is positive or negative. (**E**, **J**, and **K**) TF motif for *KLF4* (E), *MAZ* (J), and *ZNF148* (K), as sourced from JASPAR (*94*).

The gene *INO80E*, previously linked to schizophrenia through genomic studies including GWAS, transcriptome-wide association analysis, and copy number variation (CNV) analysis (*13*, *36*–*39*), emerged as a significant DE and DA risk gene in schizophrenia PRS extreme groups, specifically in excitatory neurons in superficial layers 2/3 (Exc_L2-3; Figs. 4, C and D, and 6, A to C), yet showed no association with disease status. We explored its regulatory mechanisms using a correlation-based network that included gene expression, chromatin accessibility, PRSs, and disease status to visualize the multi-omic data used in our study (Fig. 6D). The network revealed positive correlations within nodes of the same data modality but negative correlations across different node types. Notably, *INO80E* exhibited differential accessibility in Exc_L2-3 among extreme–genetic risk groups for cross-disorder and schizophrenia PRS. However, its correlation with gene expression fell below nominal significance, despite being a significant DE risk hit. Transcription factor (TF) motif enrichment analysis in *INO80E*’s promoter region identified significant *KLF4* motif enrichment (table S12 and Fig. 6E). Although *KLF4* has been associated with schizophrenia and reported to be down-regulated in patients (*40*), it was not expressed in Exc_L2-3 in our dataset.

A second gene, *HCN2*, coding for a hyperpolarization-activated cation channel crucial in pacemaker activity in the heart and brain (*33*), showed differential expression and accessibility (DE and DA risk gene) in Exc_L2-3 among extreme–genetic risk groups for schizophrenia PRS (Figs. 4, C and E, and 6, F and G), with no significant dysregulation in other cell types (Fig. 6H). The correlation-based network analysis for *HCN2* (Fig. 6I) indicated more positive correlations between data modalities than the *INO80E* network. Only with the network approach, *HCN2*’s gene scores in Exc_L2-3 were positively correlated with bipolar disorder PRS, and its expression in Exc_L2-3 showed positive correlations with other excitatory neuron populations but negative correlations with VIP and SST interneurons (In_VIP and In_SST; Fig. 6I). TF motif analysis in the *HCN2* promoter identified several significant motifs (table S12), notably for *MAZ* (Fig. 6J) and *ZNF148* (Fig. 6K), with these genes being expressed and accessible in Exc_L2-3 and most other cell types.

In summary, the network analysis emphasizes the complex interplay between genetic predisposition, cell-type–specific gene expression and chromatin accessibility in schizophrenia. Two genes were identified as significant DE and DA genetic risk hits simultaneously yet not as DE or DA genes associated with diagnosis. This finding underscores the disconnect between genetic risk and clinical diagnosis, suggesting that molecular phenotypes may provide additional insights into the biological mechanisms underlying schizophrenia.

## DISCUSSION

Our single-nucleus analysis of ~800,000 nuclei for gene expression and ~400,000 for chromatin accessibility from the OFC of 92 donors (including 57 with psychiatric diagnoses) represents a substantial increase in scale for single-cell studies in psychiatric research. Our findings revealed crucial differences in gene expression and chromatin accessibility primarily associated with genetic risk rather than diagnosis. Notably, glial cells predominantly showed molecular alterations associated with genetic risk genes, while neurons demonstrated a molecular response influenced by both diagnosis-related and genetic risk genes. Additionally, we identified distinct pathway enrichments in down-regulated genes in microglia.

In our study, most genes show differential expression in excitatory neurons between psychiatric cases and controls, aligning with prior findings (*20*). However, it is essential to consider variations in detection power among cell types when interpreting these findings. Notably, *SLIT2* and *KCNQ3*, previously linked to psychiatric disorders but without specific cell-type associations (*29*, *30*), exhibit cell-type–specific dysregulation. *SLIT2*, exclusively dysregulated in excitatory neurons’ layers 4 to 6, cluster 1, has been linked to depression- and anxiety-like behavior in mice (*29*), both of which have a substantial symptomatic overlap with bipolar disorder and schizophrenia (*41*, *42*), and the development of serotonergic and dopaminergic circuits in the forebrain (*43*). *KCNQ3*, coding for a voltage-gated potassium channel, is specifically down-regulated in microglia, suggesting a role in the excitation-inhibition imbalance and neuronal hyperexcitability implicated in bipolar disorder and schizophrenia (*30*, *44*). Its dysregulation has been associated with reduced gene expression and altered DNA methylation in bipolar disorder (*30*) and proposed as a previously unknown target in depression and anhedonia treatment (*45*). Our cell-type–specific findings expand upon previous bulk tissue studies, offering insights for more in-depth investigations into disease consequences and potential directions for therapeutic research. For instance, drugs targeting *KCNQ3* expression in microglia could modulate gene expression levels or target specific cellular pathways involved in *KCNQ3* expression to counteract disease pathology.

We examined the correlation between gene expression and chromatin accessibility to determine whether changes in expression might be influenced by alterations in chromatin. Our findings indicate that, at the cell-type level, there is generally a positive correlation between ATAC-seq signals near a gene and its expression (fig. S2, C and D), consistent with prior research (*46*). However, the relationship between gene expression and chromatin accessibility is complex and multifaceted. While chromatin accessibility plays an important role in gene regulation, it is not the sole determinant of expression levels. Specifically, of the 872 DE genes, 867 exhibited changes in gene expression without corresponding alterations in nearby chromatin accessibility. Studies like Zhang *et al.* (*47*) provide evidence of allele-specific open chromatin in the brain, suggesting that genetic risk for psychiatric disorders is often mediated through changes in chromatin accessibility. Moreover, Bryois *et al.* (*16*) highlighted that genetic risk factors can affect chromatin accessibility without necessarily causing concurrent transcriptional changes, particularly in specific neuronal and glial populations. These findings collectively suggest that gene expression and chromatin accessibility changes may operate through both independent and interconnected mechanisms in psychiatric disorders. Our H-MAGMA analysis further supports this by revealing a convergence of individual GWAS signals and overall polygenic risk in specific cell types, highlighting the importance of cell-type–specific analyses. Moreover, the observed genetic overlap between disorders underscores their interconnected nature and points toward shared molecular pathways.

Focusing on differentially accessible genes corresponding to DE genes, we identified a small subset of genes with alterations in both gene expression and chromatin accessibility between psychiatric cases and controls (*n* = 24 of 872 genes). For instance, *HES4* was consistently down-regulated in gene expression and chromatin accessibility in excitatory neurons’ layers 4 to 6, cluster 1, previously associated with abnormal psychomotor behavior in schizophrenia (*48*). Epigenetic dysregulations in *HES4* have also been shown to be related to neuronal development and neurodegeneration in postmortem brains (*49*). Similarly, *IGFBP5*, showed up-regulation in both gene expression and chromatin accessibility in OPCs, associated with depressive symptoms and cognitive dysfunction in aging (*50*). Considering that environmental factors like stress or lifestyle choices, relevant to psychiatric diseases (*51*), can influence epigenetic modifications and affect chromatin structure and gene expression (*52*), exploring these relationships becomes even more crucial. Further studies using alternative or more specific types of epigenetic regulation, such as histone modification analysis, may provide deeper insights into the regulatory mechanisms underlying psychiatric disorders.

Our analysis showed distinct regulatory patterns associated with genetic risk across disorders and specifically in bipolar disorder, MDD, and schizophrenia. These patterns differed substantially from those associated with clinical diagnosis, highlighting the importance of investigating genetic risk independently. This is crucial given the limitations of diagnosis, which may not accurately reflect the actual nature or severity of psychiatric conditions (*2*, *53*). Notably, genetic risk and clinical diagnosis exerted distinct influences on various cell lineages, particularly neuronal and glial populations, with genetic risk genes exhibiting larger effect sizes (fig. S10). Excitatory neurons exhibited significant alterations influenced by diagnosis as well as genetic risk in both ATAC-seq and RNA-seq data. This aligns with their role in synaptic and circuit-level changes often associated with psychiatric conditions (*54*). In contrast, endothelial cells and glial populations (astrocytes, OPCs, and microglia) were more distinctly influenced by genetic risk factors (76 to 97% of the genes are unique DE or DA risk genes), suggesting a more active role in genetic predisposition to psychiatric disorders (*55*). This cell-type–specific impact underscores the importance of examining diverse cell populations in psychiatric research. This distinction was not as evident in previous bulk studies, which often obscured cell-type–specific dynamics due to their aggregated nature.

Despite minimal overlap of genes between the diagnostic and genetic risk analyses, we observed correspondence on affected pathways. This observation may stem from the functional convergence of affected genes, besides a potential lack of power to detect more overlapping genes. For instance, we identified a significant enrichment of ribosomal processes in genes up-regulated in oligodendrocytes, OPCs, and endothelial cells. This finding aligns with previous research linking ribosomal dysregulation to psychiatric disorders (*56*). Dysfunction in ribosomal processes could affect key features like protein synthesis and synaptic function in psychiatric conditions (*57*). Furthermore, pathways related to neurodegenerative diseases and oxidative phosphorylation were enriched in various cell types, suggesting complex regulation across cell populations. Perturbations of protein synthesis as well as oxidative stress, usually caused by an imbalance of oxidative phosphorylation and the removal of its byproduct, can lead to excitation/inhibition imbalance implicated in the pathophysiology of schizophrenia (*56*). Specifically, the unique dysregulation pattern observed in genes down-regulated in microglia reflects their critical role in brain health (*58*), potentially linked to increased inflammation and stress-induced brain changes implicated in psychiatric disorders like schizophrenia (*59*). The ability of microglia to adapt and switch roles in response to inflammation (*60*) may underlie this observed unique dysregulation pattern, reflecting their complex and multifaceted roles in the intricate relationship between microglia, inflammation, and psychiatric conditions.

While our analysis revealed very few differentially accessible genes between cases and controls, we noticed a markedly higher number of differentially accessible genes compared to DE genes associated with genetic risk for psychiatric disorders (6418 versus 141; Fig. 4, B and C). The only genes observed as significant in both differential expression and accessibility analysis, specifically when examining genetic risk, are *INO80E* and *HCN2* in excitatory neurons’ layers 2/3 for schizophrenia. *INO80E* has been highlighted before as a promising drug target as it is a GWAS, TWAS, and CNV hit for schizophrenia (*36*), while *HCN2* has been identified as differentially methylated in the prefrontal cortex and hippocampus in schizophrenic patients (*61*, *62*), and its knockdown leads to antidepressant behavior in rodents (*63*). Together, these results suggest that chromatin accessibility alterations play a more prominent role in the genetic basis of psychiatric disorders compared to changes in gene expression levels. Chromatin accessibility may represent an earlier or more fundamental level of genetic regulation, influencing a gene’s potential for expression before actual changes in gene expression occur, as it is also the case for developmental processes in the cortex (*64*). This implies that chromatin accessibility serves as a more sensitive marker for genetic predispositions to psychiatric disorders.

Following our observation of distinct genetic risk mechanisms and diagnosis, we conducted an integrative correlation–based network analysis of the key affected genes, including *INO80E* and *HCN2*. It revealed that correlations tend to be positive within the same data modality (e.g., gene expression and PRS) and negative between different modalities. This suggests that gene expression or accessibility differences linked to genetic risk might not directly align with linear trends in genetic risk scores. This disconnect likely reflects the multifaceted nature of schizophrenia, which encompasses a spectrum of disorders with diverse clinical presentations and etiologies. Furthermore, current diagnostic criteria, while valuable, may not fully capture this underlying biological heterogeneity.

It is important to emphasize that PRS in this study capture genetic risk for a disease, which may manifest clinically in conjunction with other factors, including developmental and environmental factors. The presence of control individuals with high PRS for schizophrenia highlights this complex interplay. It is also crucial to consider the role of environmental factors, which are not captured in PRS. Exposure to stress, trauma, or infections can interact with genetic vulnerabilities to influence disease development (*65*). Future research should aim to incorporate environmental factors and longitudinal data into risk prediction models to improve their accuracy and clinical utility. Longitudinal studies can track the trajectories of genetic risk, molecular phenotypes, and clinical outcomes over time, providing a more dynamic understanding of disease progression. Integrating environmental data can help elucidate the complex interplay of genes and environment in schizophrenia.

Our network analysis revealed that genetic risk scores show a slightly stronger relationship with chromatin accessibility compared to gene expression, although these findings warrant further investigation to confirm and clarify these patterns. In exploring regulatory elements of *INO80E* and *HCN2*, our TF motif analysis identified a *KLF4* motif enrichment for *INO80E*. The absence of *KLF4* expression and accessibility in excitatory neurons’ layers 2/3 in our dataset suggests a potential mismatch in the timing of *KLF4* expression or the involvement of a different, unidentified TF. For *HCN2*, the numerous enriched motifs suggest intricate, cell-context–specific transcriptional regulation.

This study has several limitations. First, the inconsistent correlation between gene expression and chromatin accessibility suggests that other regulatory mechanisms, including trans-regulatory elements and additional epigenetic layers like DNA methylation and histone modifications, are at play. Second, our case cohort is biased toward schizophrenia diagnoses. Third, by focusing on extreme groups for genetic risk rather than treating it as a continuous variable, we applied a robust approach that has demonstrated reliability in previous studies (*10*, *66*). However, this approach may limit our ability to detect subtle effects associated with intermediate levels of genetic risk. Furthermore, the number of differentially accessible genes identified in the genetic risk analysis was substantially higher than in the case-control analysis. This likely reflects the stronger contrast and reduced heterogeneity in the genetic risk groups compared to the case-control groups. Additionally, genetic differences can directly influence open chromatin, as demonstrated by studies on chromatin QTLs (*16*). This further explains the increased number of DA genes observed in the genetic risk analysis, as it directly captures the impact of genetic variation on chromatin accessibility. Fourth, our cohort is limited to individuals of European ancestry, highlighting the need for more diverse samples in future studies. Last, the sequencing depth in our study does not reach the highest standards currently achievable.

Future research should prioritize the inference of cell-type–specific gene regulatory networks, offering a more comprehensive view of the regulatory mechanisms underlying psychiatric disorders. Higher resolution multi-omic approaches will enable more detailed insights into these mechanisms. Critically, complementing analyses of clinical diagnoses with genetic risk studies is critical for understanding disease progression and uncovering the genetic basis of psychiatric disorders. Building on the identified molecular signatures, future research should focus on developing targeted therapies or repurposing existing drugs to specifically address the underlying molecular perturbations associated with psychiatric disorders.

## MATERIALS AND METHODS

### Human postmortem brain samples

As previously described (*67*, *68*), ethics approval was obtained from both the Ludwig Maximilians-Universität (22-0523) and the Human Research Ethics Committees at the University of Wollongong (HE2018/351). Donors or their next of kin provided informed consent for brain donation. Using fresh-frozen postmortem tissues of the OFC (BA11 dissected from the third 8- to 10-mm coronal slice), sourced from the NSW Brain Tissue Resource Centre in Sydney, Australia, we conducted snRNA-seq and snATAC-seq. Our study encompassed a cohort of 92 donors. This included 35 psychiatrically healthy controls and 57 cases diagnosed with schizophrenia, schizoaffective disorder (SCA), MDD, or bipolar disorder (*n* = 38, 7, 7, and 5, respectively). The case and control groups were matched in terms of sex (38% female representation), age (means ± SD = 54.27 ± 13.64), PMI (means ± SD = 33.90 ± 14.82), and brain pH (means ± SD = 6.60 ± 0.24); see table S1.

### Nucleus isolation and snRNA-seq and snATAC-seq

Nuclei were extracted from ~50 mg of frozen postmortem brain tissue (BA11) as previously described (*67*). In short, tissue was homogenized using dounce homogenization in 1 ml nucleus extraction buffer [10 mM tris-HCl (pH 8.1), 0.1 mM EDTA, 0.32 M sucrose, 3 mM Mg(Ac)_2_, 5 mM CaCl_2_, 0.1% IGEPAL CA-630, and RiboLock ribonuclease (RNase) inhibitor (40 U/ml) (Thermo Scientific)]. Next, homogenate was layered onto 1.8 ml of sucrose cushion [10 mM tris-HCl (pH 8.1), 1.8 M sucrose, and 3 mM Mg(Ac)_2_] and ultracentrifuged at 28,100 rpm at 4°C for 2.5 hours (Thermo Scientific Sorvall WX+ 471 ultracentrifuge). Using vacuum suction, supernatant was removed, and nucleus pellet was gently resuspended in 80 μl of resuspension buffer [1× phosphate-buffered saline, 3 mM Mg(Ac)_2_, 5 mM CaCl_2_, 1% bovine serum albumin, and RiboLock RNase inhibitor (40 U/ml)]. From the same nucleus suspension snATAC libraries and snRNA libraries were simultaneously prepared using the Chromium Next GEM Single Cell ATAC Kit v1.1 and the Chromium Next GEM Single Cell 3′ Kit v3.1, respectively; following the manufacturer’s instructions. We aimed to recover 10,000 nuclei per sample for both snATAC and snRNA libraries. Libraries of the different donors were pooled equimolarly for each of the snATAC and snRNA libraries. Illumina Free Adaptor Blocking Reagent was applied as per manufacturer’s instructions. Libraries were sequenced on the NovaSeq 6000 System (Illumina, San Diego, California, USA).

### snRNA-seq data workflow

Initial processing of the snRNA-seq data, including the alignment of reads to a pre-mRNA reference (genome build GRCh38, Ensembl 98), cell barcoding, and UMI counting, was performed with Cell Ranger (cellranger count v6.0.1) (*69*). To account for substantial differences in sequencing depth between cells and samples, we downsampled reads to the 75% quantile that corresponds to 14,786 reads per cell. This downsampling procedure was performed with the downsampleReads method from the DropletUtils package v1.12.2 (*70*), brought the sequencing depth of cells in different samples to a more comparable level, and prevented biases in the analysis.

Count matrices of all donors were combined and further processed in Python (Python Software Foundation, www.python.org/), primarily using Scanpy v1.7.1 (*71*). Nuclei were filtered according to counts, minimum genes expressed, and percent of mitochondrial genes (counts < 500, genes < 300, and Mito % ≥ 15). Genes expressed in <500 nuclei were removed. One individual was filtered out because of the overall low data quality, coinciding with a low RNA integrity number (RIN) value. To ensure data integrity and the accuracy of our analysis, we conducted doublet removal using the DoubletDetection package v3.0 (*72*). Data were normalized and log transformed using sctransform v0.3.2 (*73*). Highly variable genes were identified and dimensionality reduction, including principal components analysis (PCA), and uniform manifold approximation and projection (UMAP) was performed with Scanpy (*71*). Nuclei were clustered on the basis of highly variable genes using the Leiden clustering algorithm (resolution 1.0). Four donors were filtered out because of the fact that more than 50% of their nuclei were located within one cluster, resulting in 787,046 nuclei from 87 donors; see table S2.

### snATAC-seq data workflow

The initial processing of the snATAC-seq data, including the alignment of reads to a reference (genome build GRCh38, Ensembl 98), cell calling, and count matrix generation, was performed with Cell Ranger ATAC (cellranger-atac count v2.0.0) (*74*). Further processing of the data was performed in R v4.0.5 (*75*) with the ArchR package v1.0.2 (*76*). During per-cell QC, nuclei with a transcription start site enrichment score < 4 were excluded because of a low signal-to-noise ratio. Furthermore, nuclei with less than 1000 or more than 100,000 unique nuclear fragments were filtered out. Doublet scores were inferred in ArchR (*76*), and respective doublets were removed with a filter ratio of 2.5. One donor was filtered out because of the overall low data quality, coinciding with a low RIN value. Iterative latent semantic indexing was used for dimensionality reduction to handle the high sparsity of snATAC-seq data. From this lower-dimensional space, a UMAP embedding was inferred for visualization purposes. Nuclei were clustered with resolution 1.0 via an interface to the FindClusters method from Seurat v4.0.4 (*77*), which is based on the Louvain clustering algorithm. During a final filtering, another donor with most of its nuclei clustering together and six clusters with low data quality regarding doublet scores and number of fragments were removed, resulting in 399,439 nuclei from 90 donors; see table S2.

To assess chromatin accessibility directly at the gene level, gene scores were calculated with ArchR. Gene scores are estimates of gene expression predicted from the accessibility of the regulatory region surrounding a gene (100 kb up- and downstream), whereby the signal is weighted by the distance to the gene (*76*).

### Cell-type assignment of snRNA-seq and snATAC-seq data

An initial cell-type assignment to clusters of nuclei in the snRNA-seq data was carried out using a label transfer algorithm [scArches v0.4.0; (*78*)/scANVI; (*79*)]. Thereby, cell-type labels were adopted from a dataset of the Allen Brain Map covering six distinct regions of the human cortex [Human Multiple Cortical Areas; (*80*)] to our snRNA-seq dataset, using a variational inference model. Each cluster was labeled with the cell type assigned to the majority of nuclei within this cluster. Subsequently, the cell-type labels were fine-tuned through manual curation on the basis of the expression of known marker genes, as previously described (*67*). Marker genes included the following: astrocytes: *AQP4*, *CLU*, *GFAP*, and *GJA1*; endothelial: *CLDN5*, *COBLL1*, *FLT1*, and *SYNE2*; excitatory neurons: *SATB2*, *SLC17A6*, and *SLC17A7*; inhibitory neurons: *GAD1*, *GAD2*, *NXPH1*, and *SLC32A1*; microglia: *APBB1IP*, *C3*, and *P2RY12*; oligodendrocytes: *MPB*, *MOBP*, *PLP1*, and *RNF220*; OPCs: *OLIG1*, *OLIG2*, *PDGFRA*, and *VCAN*; and astrocyte subtypes: higher *GFAP* and *ARHGEF4* expression [fibrous astrocytes (Astro_FB)] versus higher expression of *ATP1A2*, *GJA1*, and *SGCD* [protoplasmic astrocytes (Astro_PP)] (*81*). Excitatory neuron subtypes were labeled on the basis of the expression of cortical-layer specific marker genes: layers 2 and 3: *CUX2* and *RFX3*; layer 4: *IL1RAPL2*, *CRIM1*, and *RORB*; and layers 5 and 6: *RXFP1*, *TOX*, *DLC1*, and *TLE4* (*19*, *81*). Inhibitory neuron subtypes were labeled on the basis of the expression of interneuron markers *LAMP5*, *PVALB*, *RELN*, *SST*, and *VIP*. PVALB inhibitory neurons consisted of two subtypes: basket cells (In_PVALB_Ba) and chandelier cells (In_PVALB_Ch; identified on the basis of the high expression of *RORA*, *TRPS1*, *NFIB*, and *UNC5B*) (*82*).

For the initial assignment of cluster identities in the snATAC-seq data, the data were integrated with snRNA-seq data in ArchR (*76*) via a parallelized interface to the FindTransferAnchors function in Seurat (*77*). Nuclei from snATAC-seq are getting aligned with nuclei from scRNA-seq by comparing the gene score matrix with the gene expression matrix. Each snATAC-seq nucleus is labeled with the cell type of the most similar scRNA-seq nucleus. Adjacently, cluster identities were refined manually on the basis of gene scores of the marker genes mentioned above. Although known marker genes of endothelial cells did not exhibit distinct gene scores in the cluster labeled as endothelial cells, the cluster’s clear separation of other clusters, the unambiguous assignment as endothelial cells via label transfer, and imputed gene scores allowed for a confident assignment of the cluster as endothelial cells.

### Pseudobulk replicates of snRNA-seq and snATAC-seq data

To enable downstream analyses that require replicates with measurements of statistical significance, such as peak calling on ATAC-seq data or differential testing on either data modality, pseudobulk replicates were created. Specifically, gene expression and chromatin accessibility count matrices were summed up from the cells within each cell-type–donor pair, creating pseudobulk replicates resembling bulk RNA-seq and ATAC-seq data per cell type.

The pseudobulk replicates used for cell-type–specific peak calling were generated with an ArchR method summarizing multiple sufficiently similar donors within a cell type to circumvent sparsity. Because such multi-individual pseudobulk replicates are not suitable for our downstream analyses, the ArchR-generated replicates were only used during peak calling.

### Peak calling on snATAC-seq data

Peak calling was performed per cell type in ArchR (*76*) on the basis of pseudobulk replicates via an interface to MACS2 (*83*). To facilitate downstream computation, peaks have a fixed width of 501 base pairs and are merged across pseudobulk replicates and cell types via a ranking of peaks by normalized significance and the iterative removal by overlap. The resulting matrix contains a single merged peak set of fixed-width peaks.

### DNA extraction, SNP genotyping, and imputation

From 10 mg of brain tissue, genomic DNA was isolated using the QIAamp DNA mini kit (QIAGEN) according to manufacturer’s instructions. Following extraction, DNA samples were concentrated using the DNA Clean & Concentrator-5 (Zymo Research).

Samples were genotyped with Illumina GSA-24v2-0_A1 arrays, following the manufacturer’s protocols (Illumina Inc., San Diego, CA, USA). QC was performed in PLINK v1.90b3.30 (*84*). Sample QC included removal of donors with a missing rate > 2%, as well as cryptic relatives [proportion of identity-by-descent alleles (PI-HAT) > 0.125]. Donors with autosomal heterozygosity deviation (|Fhet| > 4 SD) and genetic outliers (distance in ancestry components from the mean > 4 SD) were also excluded. Variants with a call rate < 98%, a minor allele frequency (MAF) < 1%, and *P* values from the Hardy-Weinberg equilibrium (HWE) test ≤ 10^−6^ were removed during variant QC. Imputation was conducted using shapeit2 (*85*) and impute2 (*86*), making use of the 1000 Genomes Phase III reference sample. Imputed single-nucleotide polymorphisms (SNPs) with an INFO score below 0.6, MAF < 1%, or deviation from HWE (*P* value <1 × 10^−5^) were excluded from further analysis, resulting in a final set of 9,652,209 SNPs in 92 donors.

### Calculation of PRSs

Summary statistics of GWASs for a cross-disorder phenotype (*4*), schizophrenia (*7*), MDD (*8*), bipolar disorder (*9*), and height (*31*) (as a nonpsychiatric control) were used to calculate PRSs. Posterior effect sizes were inferred from the GWAS summary statistics using PRS-CS v1.0.0 (*87*). The linkage disequilibrium reference panel used was the one based on the European samples of the 1000 Genomes Project phase 3, as accessible on the PRS-CS GitHub page. For schizophrenia, a highly polygenic trait, we set the global shrinkage parameter (phi) of PRS-CS to 0.01, while no specific phi parameter was specified for the other traits, given the larger sample size of the GWASs, allowing phi to be derived from the data. PRSs per donor were calculated from the previously inferred posterior effect sizes in PLINK v2.00a2.3LM (*84*) with the score parameter.

### Definition of disease status for differential testing

Differential expression (DE) and differential accessibility (DA) was tested between all donors with a psychiatric diagnosis (schizophrenia, SCA, bipolar disorder, or MDD) against all donors in the control group. Psychiatric disorders were analyzed as a cross-disorder phenotype due to their shared genetic risk, overlapping symptomatology (*2*–*4*), and the high degree of diagnostic comorbidity observed across these conditions. For instance, a study has shown that most individuals with psychiatric disorders receive more than one diagnosis during their lifetime (*88*). This shared biological and clinical foundation increases statistical power of the analyses and enables the identification of shared molecular dysregulations and underlying pathways. Nevertheless, we note that our sample is biased toward schizophrenia, with a higher representation of donors diagnosed with schizophrenia compared to other psychiatric disorders, which should be considered when interpreting our findings. Because of this skewed distribution of diagnoses, we also performed a DE analysis on a schizophrenia-specific subsample, which is detailed in the supplementary material.

### Definition of groups for testing between high and low genetic risk

To assess DE and DA also with regard to overall genetic predisposition, differential testing was performed between donors with high and low genetic risk for a trait or disease. Acknowledging the consensus within the research community that reliable risk predictions are most feasible at the extreme ends of the PRS distribution (*10*, *66*), we categorized genetic risk into binary groups representing these extremes rather than treating it as a continuum. Specifically, we selected 20 donors with the highest PRS and 20 donors with the lowest PRS within the cohort for each trait or disease.

Subsequently, we used propensity score matching to identify subsets of extreme groups that are matched on the basis of key covariates such as age, sex, brain pH, PMI, and RIN (Fig. 4A). Sex was matched exactly (fig. S7). We used the matchit function from MatchIt v4.5.5 (*89*) for this purpose. The resulting number of donors in each extreme group ranges from 11 to 17, with specific counts being 17 donors for both high- and low-PRS groups in cross-disorder, 13 donors for both groups in schizophrenia, 14 donors for both groups in bipolar disorder, 11 donors for both groups in MDD, and 14 donors for both groups in height. For each trait or disease, both extreme groups include psychiatric cases and controls (table S5), given that a high or low genetic risk does not imply the progression or protection of a disease.

### Selection of covariates for differential testing between disease status and extreme–genetic risk groups

To comprehensively evaluate the impact of biological variables and batch effects on the data and to select relevant covariates for differential testing, we assessed the impact of potential confounders on the RNA-seq data. Given the assumption that technical covariates remain consistent across cell types, a full pseudobulk count matrix was created by summing gene-wise counts across all cell types. Only genes with a minimum of 10 counts in at least 90% of the samples were retained for the covariate selection process. Data were normalized with the variance stabilizing transformation in DESeq2 (*90*), and PCA was applied. A significant correlation between continuous variables and one of the first 10 principal components (PCs) was observed for RIN, PMI, pH, and age. Further exploration using canonical correlation analysis identified the library preparation batch (lib_batch) as a covariate. However, the inclusion of the library preparation batch into the model was limited to disease status, owing to the insufficiency of observations within each batch in the genetic risk model to support a categorical variable in the genetic risk model. Additionally, we included sex as a commonly known confounder as a covariate into our model.

To account for hidden noise, PCA was performed after having normalized and transformed the data and regressed out the effect of all mentioned covariates and our variable of interest [disease status (Disease_Status) or genetic risk group (Genetic_Risk), respectively] of the data using voom and removeBatchEffect from the limma package v3.56 (*91*). We included the first PC (PC_noise) as additional covariate into our final model for differential testing: (~Disease_Status/Genetic_Risk + Sex + Age + pH + RIN + PMI + lib_batch + PC_noise). RIN was not present for one donor and therefore imputed to the median value across the cohort.

To keep analyses consistent and due to the fact that snRNA-seq and snATAC-seq data were generated from the exact same tissue and that library preparation was performed in the same batches for both data modalities, the same covariates, except RIN, were included into the final model of differential chromatin accessibility analysis.

### Differential expression analysis

DE was tested on the pseudobulk level with DESeq2 v1.40.2 (*90*). For each cell-type–specific count matrix, genes were filtered for a minimum of 10 counts in 75% of the pseudobulk samples. After data normalization with the variance stabilizing transformation in DESeq2 (*90*), outlier samples were excluded by iterative PCA and the removal of samples with a distance of more than 3 SDs from the mean on the first PC (detailed numbers of retained samples in table S6). We tested for DE with DESeq2 using the Wald test. Genes with a FDR ≤ 10% were reported as significant, given that the pseudobulk approach is considered more conservative than single-cell DE methods (*92*).

### Differential chromatin accessibility analysis

DA was tested on the pseudobulk level for each cell type using gene scores. As gene scores do not follow the typical characteristics of count data, differential testing was not performed with DESeq2. Pseudobulk gene scores were normalized by the number of cells aggregated per pseudobulk sample, and outliers were filtered the same way as during DE analysis (detailed numbers of retained samples in table S6). Genes exhibiting scores above 0.1 in less than 75% of the samples were filtered out and removed from further analysis. After fitting a linear model including the previously described covariates, we confirmed that the residuals of the model followed a normal distribution. A Wald test was performed, and log_2_ FCs were calculated. Genes with FDR ≤ 10% were considered as significant.

### Differential risk group analysis

Differential risk group analyses of gene expression (DE risk analysis) and chromatin accessibility (DA risk analysis), comparing donors in high– and low–genetic risk groups for a phenotype, were performed analogously to differential testing between cases and controls. Gene filtering, normalization, and outlier removal followed the same principles (detailed numbers of retained samples in table S6), and genes with FDR ≤ 10% were considered significant.

### Pathway enrichment analysis

Pathway enrichment analysis was conducted with clusterProfiler v4.8.1 (*93*). The 250 genes with the most significant up- and down-regulation for each cell type according to FDR values were assessed for overrepresentation of KEGG pathways. The choice of not only testing the significant DE and DA genes was made to make this analysis comparable between cell types. Two hundred fifty genes per direction of regulation correspond to about 50% of the number of DE genes in Exc_L2-3, the cell type with the highest number of DE genes between cases and controls. Any KEGG pathway significant in at least one cell type (FDR ≤ 0.05) is shown in the respective heatmaps. To summarize single KEGG pathways in categories, a hierarchy of KEGG pathways was downloaded from the KEGG Pathway Database (www.genome.jp/kegg/pathway.html, accessed 19 June 2023) and used to annotate the enrichment heatmap.

### TF motif enrichment analysis

To assess whether peaks in the promoter regions of a given gene are enriched for binding sites of specific TFs, a TF motif enrichment analysis was performed within the ArchR framework. As an initial step, the addMotifAnnotation function was used to obtain binary information for each peak-TF pair whether a respective motif is present in the peak or not. TF motif information was obtained from the JASPAR 2020 database (*94*). Subsequently, an adapted version of the peakAnnoEnrichment was applied to test the peaks in a given gene’s promoter region for enriched presence of TF motifs compared to the presence in all peaks using a hypergeometric test. TF motifs with an adjusted *P* value ≤ 0.05 were reported as significantly enriched.

### Comparison to previous findings

We conducted a comparative analysis of DE results for disease status against previously documented cell-type–specific transcriptomic changes in the prefrontal cortex of patients with schizophrenia. This comparison aimed to evaluate the reproducibility of our findings in relation to other studies. Effect sizes of the DE analysis based on the complete cohort, as well as the analysis based on schizophrenia cases only, were correlated with those reported in a scRNA-seq meta-analysis by Ruzicka *et al.* (*20*) (sample sizes, 140; cell counts, 469,000). For each cell-type pair, we calculated Pearson’s correlation coefficient to measure the relationship between effect sizes for all genes examined in both studies.

### Network inference

For given genes that are DE and accessible between extreme–genetic risk groups for schizophrenia, correlation-based networks were inferred to integrate gene expression and chromatin accessibility across the different cell types as well as disease status and PRS for the aforementioned disorders and traits. The analysis was based on the donors that are part of the extreme–genetic risk groups for schizophrenia. Gene expression and gene score levels were normalized and corrected for sex, age, RIN, PMI, pH, and the library preparation batch. Spearman correlation was calculated between each pair of features. Correlations with a nominal *P* value ≤ 0.05 are shown in each network, and edge strength/weight is represented by the absolute value of the correlation coefficient, with higher values indicating stronger correlations. The network was visualized using the R package ggnetwork v0.5.10 (*95*), where stronger correlations result in thicker edges between nodes.
