# Supplementary material for: Contrasting genetic predisposition and diagnosis in psychiatric disorders: A multi-omic single-nucleus analysis of the human OFC
Source: Sci Adv. 2025 Mar 7;11(10):eadq2290. doi: 10.1126/sciadv.adq2290 (PMC11887846; doi:10.1126/sciadv.adq2290)
Supplement: Supplementary file 1 — Supplementary Text Figs. S1 to S11 Legends for tables S1 to S15 [file sciadv.adq2290_sm.pdf]

Science Advances
AAAS

Supplementary Materials for

Contrasting genetic predisposition and diagnosis in psychiatric disorders: A multi-omic single-nucleus analysis of the human OFC

Nathalie Gerstner *et al.*

Corresponding author: Janine Knauer-Arloth, arloth@psych.mpg.de

Sci. Adv. **11**, eadq2290 (2025)
DOI: 10.1126/sciadv.adq2290

The PDF file includes:

Supplementary Text
Figs. S1 to S11
Legends for tables S1 to S15

Other Supplementary Material for this manuscript includes the following:

Tables S1 to S15

Supplementary Text

Supplementary Methods

1. Correlation analysis between gene expression and chromatin accessibility

The assessment of the number of peaks nearby each gene and the number of nearby correlated peaks per gene was performed for each cell type separately on the pseudobulk level. Peaks with less than 5 counts in more than 50% of the samples were removed from the peak matrix and genes with less than 5 counts in more than 75% were excluded from the count matrix. The less stringent filtering in the peaks was applied due to the even sparser signal in ATAC-seq data. Gene expression and peak matrix were normalized with the variance stabilizing transformation in DESeq2 (*91*). Peaks within a 100 kb window from the gene body, the default distance used to calculate gene scores in ArchR (*77*), were considered to be nearby a gene and tested for correlation. Pearson's correlation coefficient was used as a measure of the association between gene expression and chromatin accessibility.

In addition to the correlation between gene expression and chromatin accessibility on the peak level, expression levels were also correlated with gene scores. This analysis was performed in each cell type separately and across all cell types. On the pseudobulk level, Pearson's correlation coefficients were calculated across all genes between expression and gene scores averaged across all samples. Additionally, the distribution of Pearson's correlation coefficients calculated between gene expression and gene scores across all pseudobulk samples for each gene was compared to a random distribution obtained by correlating gene expression with a random permutation of gene scores.

2. Downsampling of nuclei

To dissect to what extent the number of DE genes in a cell type is influenced by its nuclei count and consequently the number of genes tested for differential expression, a downsampling analysis was performed. The nuclei per cell type were downsampled to the 75%, 50% and 25% percentiles of nuclei (40,793, 31,504, 14,416 nuclei respectively). Differential expression analysis was performed on the downsampled datasets as described in Methods. In a separate analysis, we included nuclei count as a covariate in the DE model to evaluate its specific impact on the results.

3. Differential expression analysis in schizophrenia subsample

To investigate the extent to which dysregulation signals between cross-disorder cases and controls are influenced by schizophrenia, which is the most prominent diagnosis in the cohort, additional subsampling and differential expression analyses were performed. The cohort was first

restricted to schizophrenia cases and controls, and differential expression analysis was conducted following the methodology used in the primary analysis (Methods 2.5.3-2.5.4). Additionally, a stratified subsample of the cross-disorder cohort was generated, maintaining the same distribution of diagnoses as the original cohort but with a sample size equivalent to the schizophrenia-only subsample. Differential expression analysis was then carried out on this stratified subsample to determine whether differences observed between the schizophrenia and cross-disorder analyses could be attributed to reduced sample size and statistical power or represented distinct biological signals. Effect sizes from the primary cross-disorder analysis (CDall) were correlated with those from the schizophrenia-only analysis (SCZ) and the stratified cross-disorder subsample analysis (CDsub) for each cell type. These correlations were used to evaluate the overlap and differences between the three analyses.

4. GWAS enrichment analysis

GWAS enrichment analysis was performed with H-MAGMA v1.10 (*36*). A mapping of SNPs to genes was generated based on GWAS summary statistics for schizophrenia (*7*), bipolar disorder (*9*) and MDD (*8*) and the european 1,000 genomes reference panel downloaded from the H-MAGMA github page (https://github.com/thewonlab/H-MAGMA). Based on these results, a gene-level analysis in the form of a gene property analysis was performed with the "--gene-covar" argument in MAGMA. This analysis allows the input of a continuous variable (here: DE (risk) results in the form of $-\log_{10}(P)*\log_2(\text{fold change})$) into the gene-level regression framework to test if DE related to disease status/genetic risk is associated with GWAS results.

Supplementary Results

1. Cell type-specific cis-regulatory gene regulation

To elucidate the specific cis-regulatory interactions between chromatin accessibility and gene expression within distinct cell types, independent of any disease phenotype influence, we conducted a thorough analysis. This involved quantifying the number of proximate peaks (within 100 kb of the gene body) for each gene. Subsequently, we correlated the signal of these peaks with gene expression levels after applying appropriate filtering and normalization techniques specific to each cell type.

For example, in oligodendrocyte precursor cells (OPCs), the range of nearby peaks within the 100 kb region surrounding the gene body ranges from 0 to 112, with a median count of 6. More than 1,500 genes exhibited no proximate peaks in their vicinity (Fig. S2A). While similar patterns emerged for other cell types, they displayed distinct maximum values, consistently low median counts, and a substantial number of genes lacking nearby peaks within the 100 kb region from the gene body. Among the peaks situated near a gene, even fewer demonstrated a

significant correlation with the respective gene's expression levels. The maximum number of peaks showing nominal significance ($P \leq 0.05$) was 18, while nearly 8,000 genes were without any correlated peaks (Fig. S2B).

Due to this sparse signal on the peak level, we examined the relationship between gene expression and chromatin accessibility on the gene level, making use of gene scores which predict the level of gene expression from the accessibility of gene regulatory elements nearby a gene without the necessity to call peaks. The correlation between the mean normalized gene expression values and gene scores across donors significantly correlates across (R = 0.47, Fig. S2C) and within cell types (R = [0.4, 0.56] in all cell types, Fig. S2D). While the correlation between normalized gene expression and gene scores remains high if we correlate the respective pseudobulk samples across all cell types (Fig. S2E), thereby keeping cell type and sample-specific differences in the data, correlations are rather low and partly even negative if we correlate only pseudobulk samples within a specific cell type, thereby keeping only sample-specific differences (Fig. S2F). However, the distribution of correlations is still significantly different from a random distribution, generated with a permutation of the gene scores across pseudobulk samples.

As a result, we opted to conduct downstream analyses at the gene score level. This approach addresses the challenge of missing (and correlated) peaks for numerous genes, providing a more comprehensive view of the regulatory landscape surrounding each gene.

2. Differences in detection power between cell types

The number of DE genes in each cell type was influenced by its nuclei count (Fig. S3A) and consequently the number of genes tested. Downsampling the nuclei per cell type to the 75%, 50% and 25% percentiles of nuclei (n=40,793, 31,504, 14,416 respectively), revealed that the gap between the number of tested genes and DE genes in excitatory neurons and other cell types becomes smaller with the level of downsampling, but excitatory neurons still exhibited the highest number of DE genes (Fig. S3B-C).

To further evaluate the impact of nuclei count, we performed a separate differential expression analysis with nuclei count included as a covariate (Table S13). This analysis showed that, particularly in Exc_L2-3 and oligodendrocytes, several genes initially identified as DE no longer met the significance threshold (Fig. S3D). This outcome is expected, as introducing an additional covariate adds a degree of freedom to the model, influencing significance thresholds. The distribution of FDR values for genes no longer classified as DE shows that many of these genes remained close to the significance threshold, suggesting that their exclusion was not due to drastic shifts in the underlying data but rather subtle changes resulting from the inclusion of nuclei count as a covariate.

3. Differential gene expression in schizophrenia subcohort

The comparative analysis revealed distinct patterns in the correlation of differential expression results across the schizophrenia-specific cohort, the full cross-disorder cohort, and the stratified subsampled cross-disorder cohort (DE hits for subsamples in Table S14-S15). For 13 of the 19 cell types, the schizophrenia-specific results were more strongly correlated with the full cross-disorder cohort than with the stratified subsampled cohort. Conversely, 6 cell types showed a stronger correlation between the subsampled cross-disorder cohort and the full cross-disorder cohort (Fig. S3E). These findings highlight a dual effect: certain signals appear to be primarily driven by schizophrenia diagnoses, while others reflect broader, shared effects across multiple psychiatric disorders. This observation is consistent with the substantial diagnostic overlap documented in psychiatric research, where the majority of patients are diagnosed with multiple conditions during their lifetimes (*89*). Such comorbidity underscores the interconnected molecular and pathological basis of psychiatric disorders and supports the utility of a cross-disorder analytic framework, despite the unequal distribution of diagnoses in the cohort.

4. GWAS enrichment analysis using H-MAGMA

To ascertain whether DE risk genes for specific traits and cell types are enriched for GWAS-associated genes for psychiatric disorders (bipolar disorder (*9*), MDD (*8*) and schizophrenia (*7*)), we conducted a GWAS enrichment analysis using H-MAGMA (*36*). No significant enrichments of GWAS-associated genes emerged among the DE risk results for cross-disorder phenotype, bipolar disorder, MDD and height. However, we identified significant enrichments of schizophrenia GWAS-associated genes in the DE risk results for schizophrenia within basket cells (In_PVALB_Ba), excitatory neurons layers 2 to 3 (Exc_L2-3) and endothelial cells (Fig. S8F). Similarly, MDD GWAS-associated genes exhibited significant enrichment in endothelial cells' DE risk results for schizophrenia (Fig. S8F).

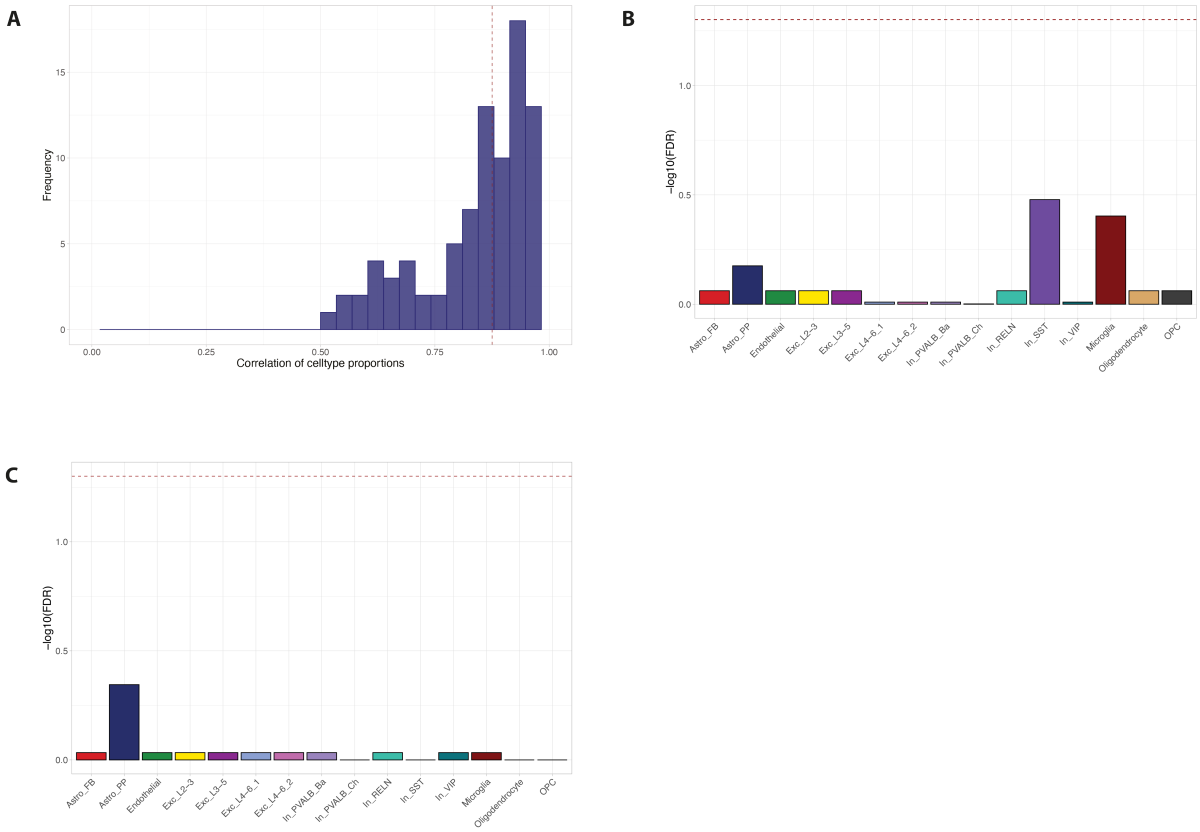

Figure S1. Differences in cell type proportions between data modalities and disease status. (A) Histogram of Pearson correlation coefficients between cell type proportions in snRNA-seq and snATAC-seq data across all donors. (B-C) Significance of differences in cell type proportions between snRNA-seq cases and controls (B) and snATAC-seq cases and controls (C). Height of the bar represents -$\log_{10}$-transformed FDR values of Wilcoxon rank-sum test and the dashed red line corresponds to the FDR cutoff of 0.05.

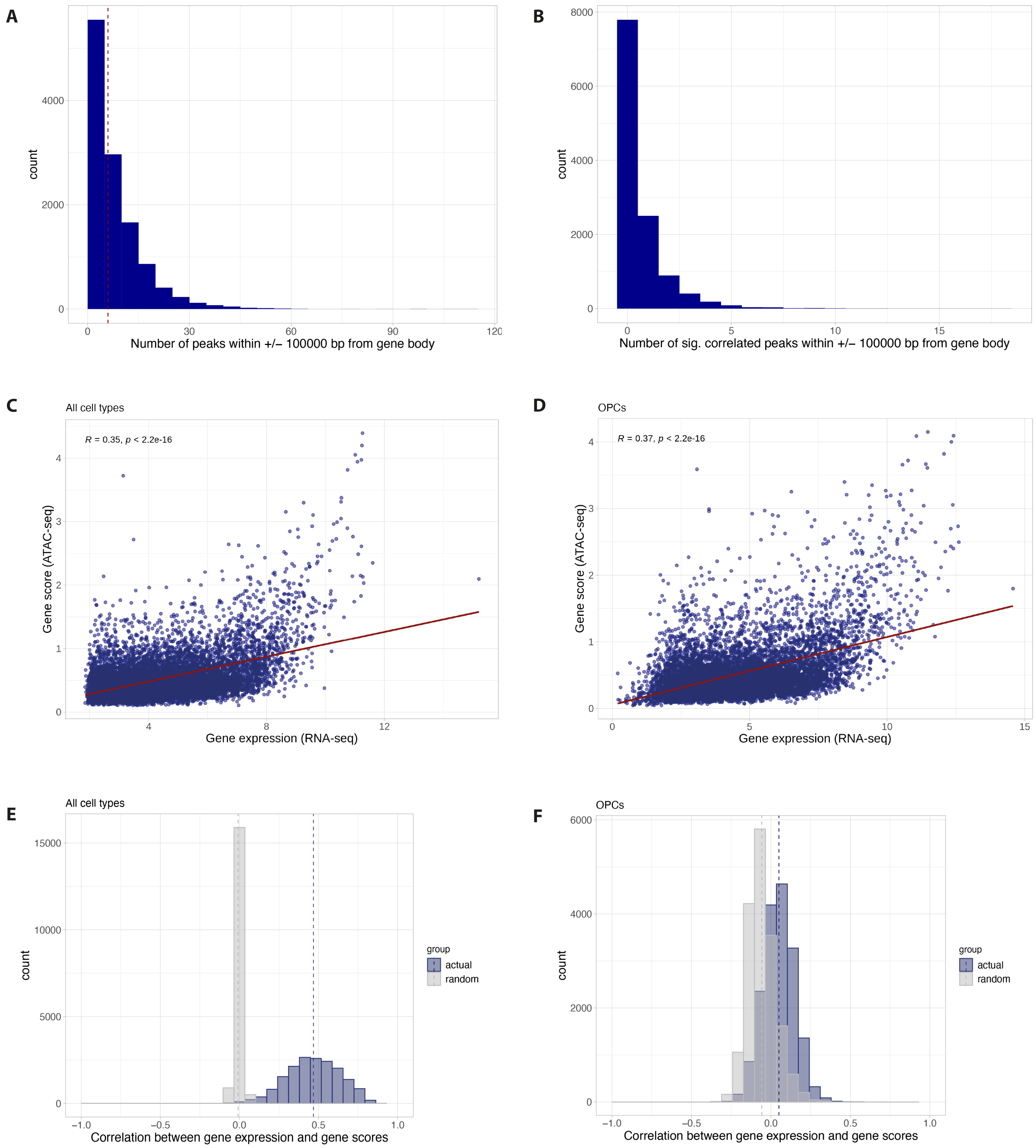

Figure S2. Cis-regulatory interactions between chromatin accessibility and gene expression. OPCs are chosen as an exemplary cell type in this figure. (A) Histogram of the number of peaks within a 100kb window from the gene body for all genes tested for differential expression in OPCs. Dashed red line indicates the median number of peaks. (B) Histogram of the number of nominally significantly correlated peaks ($P \leq 0.05$) within a 100kb window from the gene body in OPCs. (C-D) Mean gene expression levels plotted against mean gene score levels across all cell types (C) and in OPCs (D). The red line represents a linear model fitted on the data. Pearson correlation is shown in the upper left corner. (E-F) Histogram of correlations between gene expression and gene score levels on the donor level across all cell types (E) and in OPCs (F). The distribution of the actual correlation coefficients (blue) is plotted along the distribution obtained by randomly permuting the donor levels (gray). The dashed line indicates the mean values respectively.

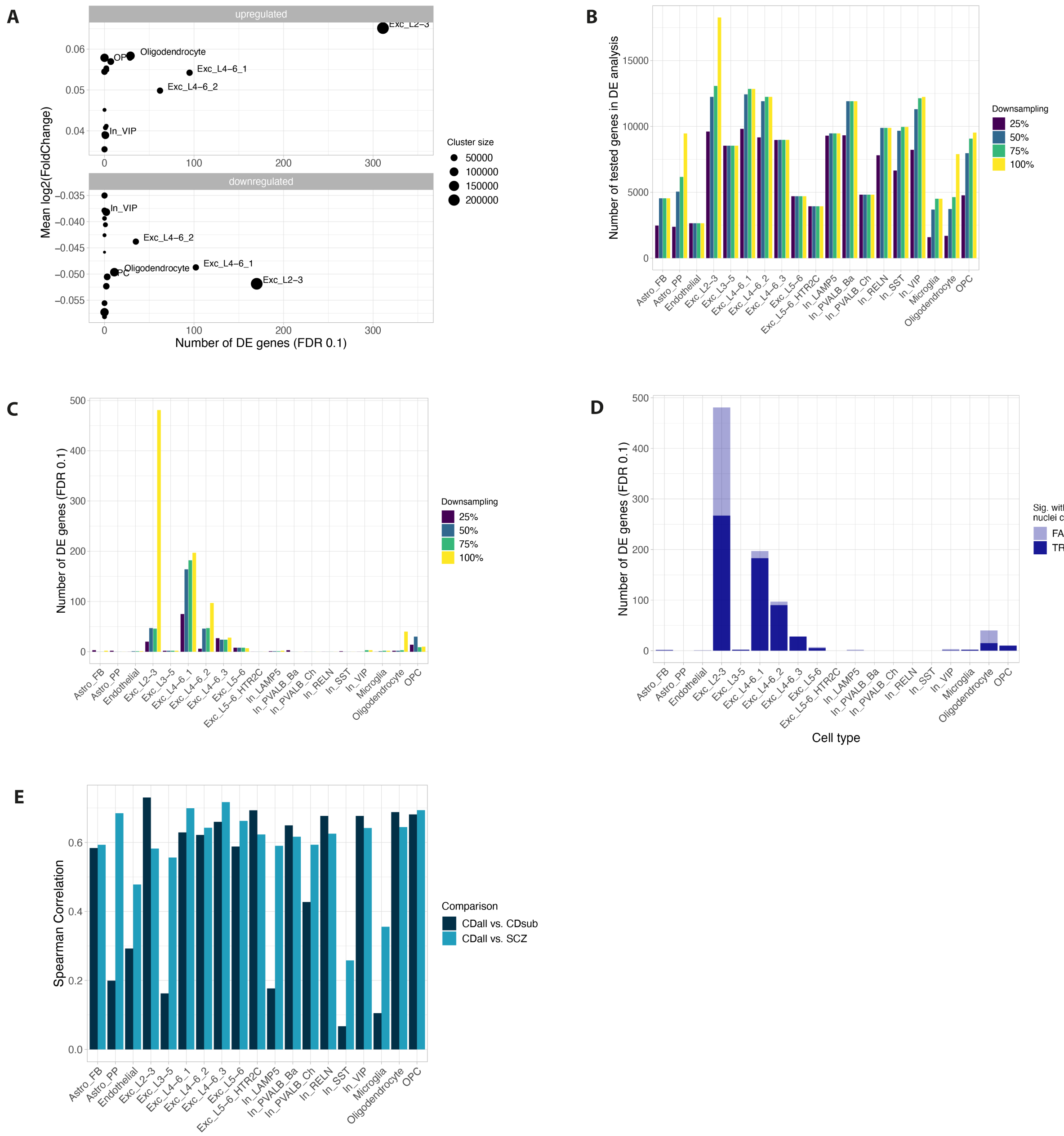

Figure S3. Transcriptional alterations between psychiatric cases and controls. (A) Number of DE genes (FDR ≤ 0.1) plotted against the mean log$_2$-fold change for up- and downregulated genes separately. Dot size indicates the cluster size. (B-C) Barplot representing the number of genes tested for differential expression (B) and the number of significant DE genes (C) using the full dataset and datasets downsampled to the 75%, 50% and 25% percentile of nuclei per cell type which is indicated by color. (D) Barplot visualizing the proportion of DE genes that is also significant using the model with nuclei count as a covariate. (E) Barplot visualizing the Spearman correlation coefficients of effect sizes across cell types between the differential expression results on the full cohort (CDall), a subset of the full cohort stratified for diagnoses (CDsub), and a subset only including schizophrenia cases and controls. Dark blue represents the correlation between the full cohort and the stratified subsampled cross-disorder cohort, while light blue represents the correlation between the full cohort and the schizophrenia-specific subset of the cohort.

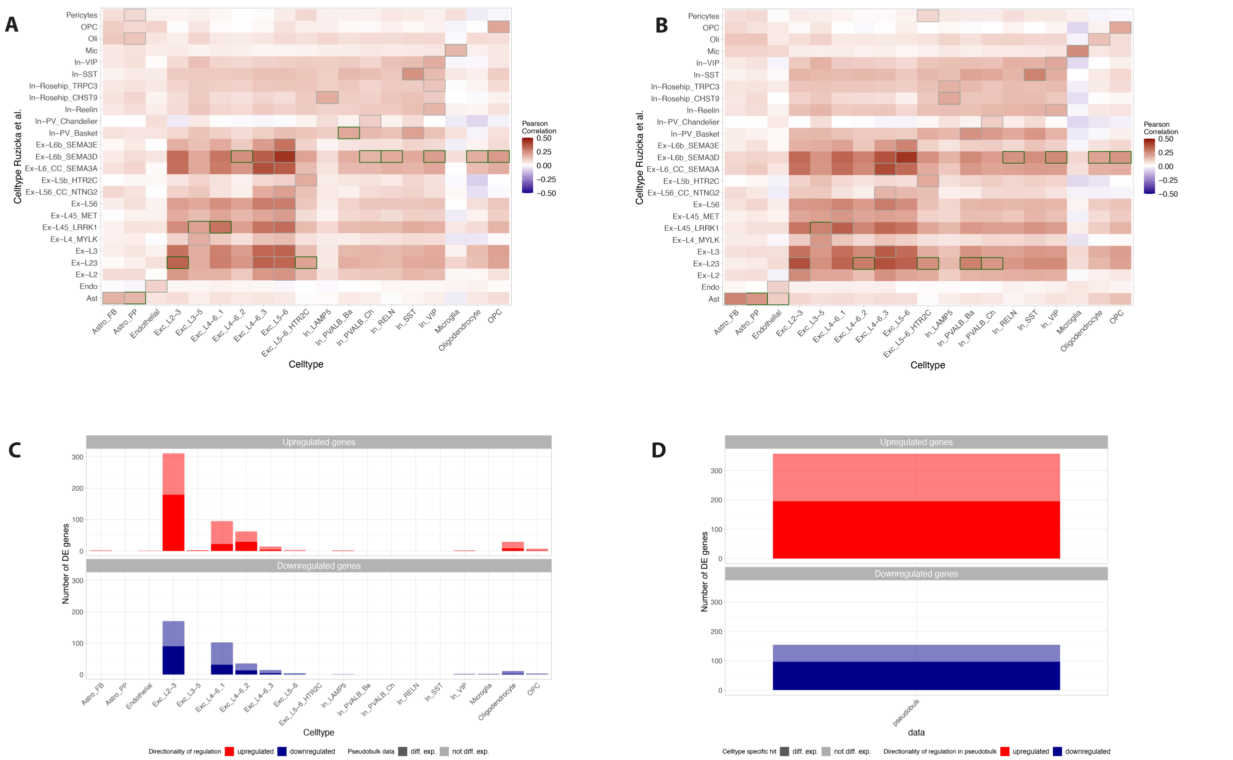

Figure S4. Comparison of DE results with prior research and full pseudobulk analysis. (A-B) Correlation analysis between effect sizes from our study based on the complete cohort (A) and the schizophrenia-only subcohort (B) and effect sizes reported in Ruzicka et al. (*20*) for each pair of cell types. Color indicates the Pearson correlation coefficient based on the effect sizes for the shared set of genes tested in the respective cell types. Dark green borders mark the highest correlation per column, while gray borders mark the highest correlation per row. (C) Barplot representing the number of DE genes per cell type for up- and downregulation separately and the proportion of genes also identified as DE based on full pseudobulk data, represented by darker color. (D) Barplot representing the number of DE genes based on full pseudobulk data for up- and downregulation separately. The darker parts of the bars represent the proportion of genes also identified as DE in at least one cell type.

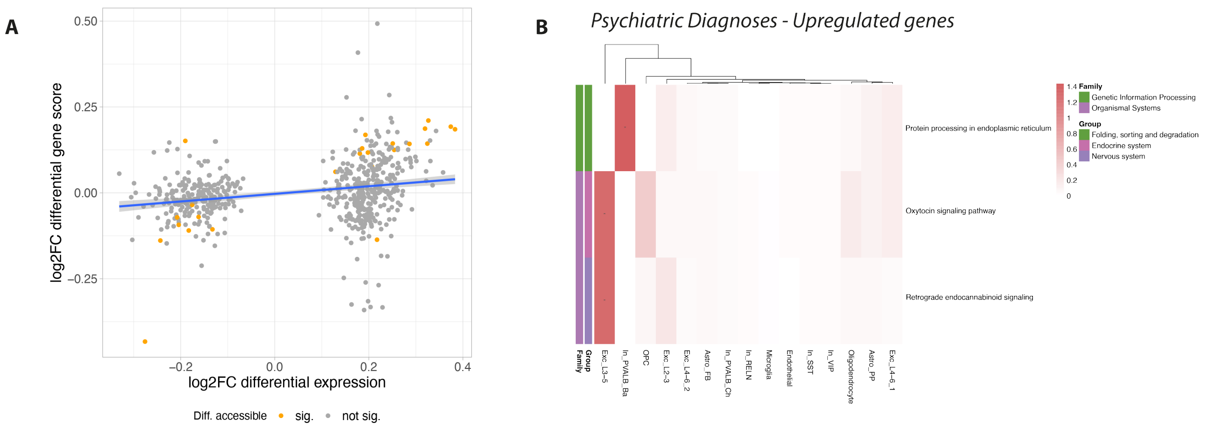

Figure S5. Epigenomic alterations between psychiatric cases and controls. (A) Log_2-fold changes of differential expression and accessibility analysis for all DE genes across cell types plotted against each other with significance in the same cell type indicated by color. The blue line represents a linear model fitted on the data. (B) Results of KEGG pathway enrichment analysis for 250 most up- and downregulated genes per cell type. All pathways significantly enriched in at least one cell type are included into the heatmap. Color represents -log10-transformed FDR values and asterisks indicate significance (FDR $\leq$ 0.05). Colored annotations of the pathways on the left side of each plot indicate to which pathway group and family a pathway belongs. The dendrograms visualize k-means clustering of cell types according to enrichment results.

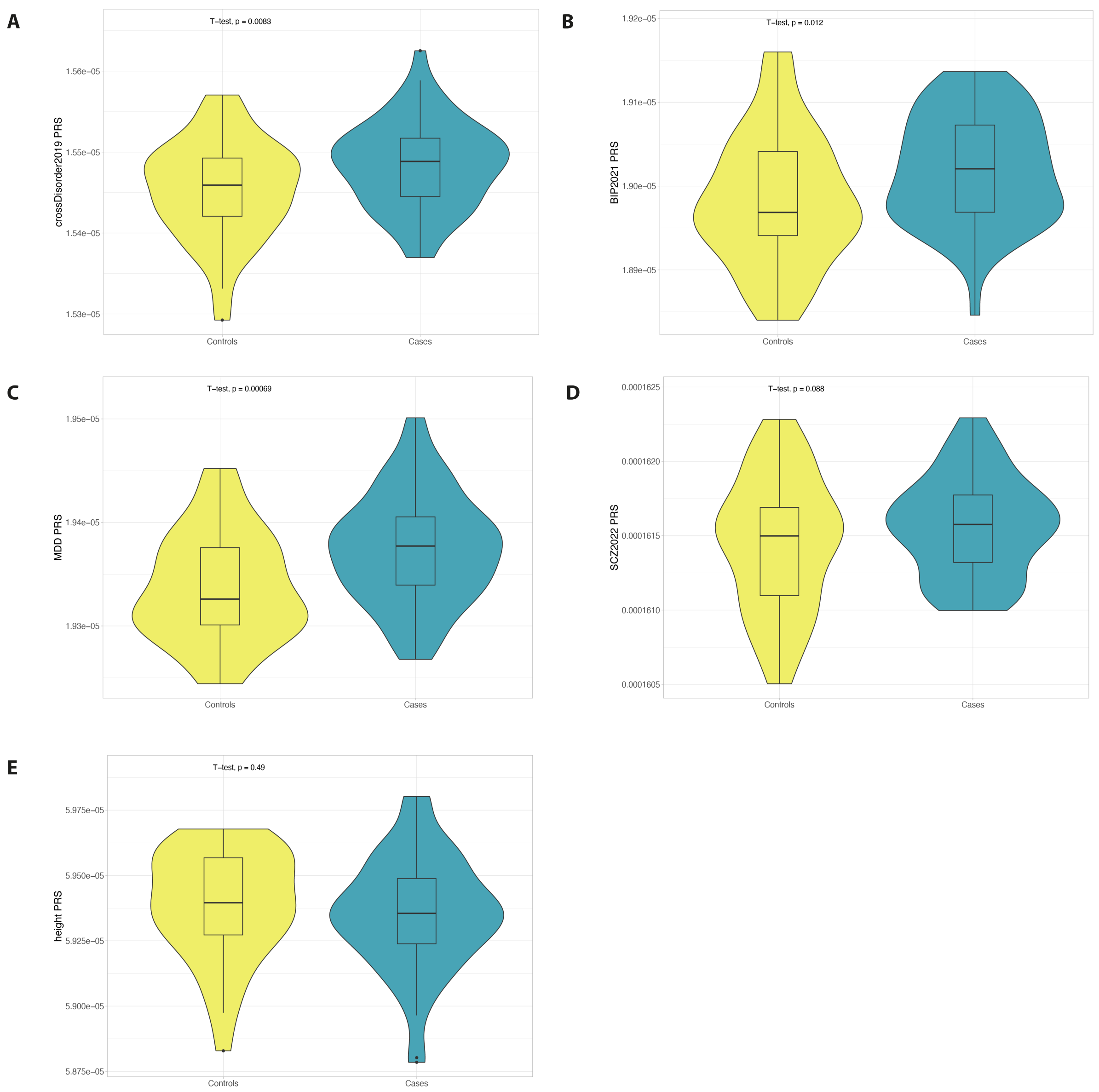

Figure S6. Genetic risk for psychiatric disorders in cases and controls. (A-E) Distribution of polygenic risk scores (PRS) for cross-disorder phenotype (A), bipolar disorder (B), MDD (C), schizophrenia (D) and height (E) for controls and cases. A one-sided t-test was used to test for differences in cross-disorder, bipolar disorder, MDD and schizophrenia PRS between cases and controls, while a two-sided t-test was used to test for differences in height PRS.

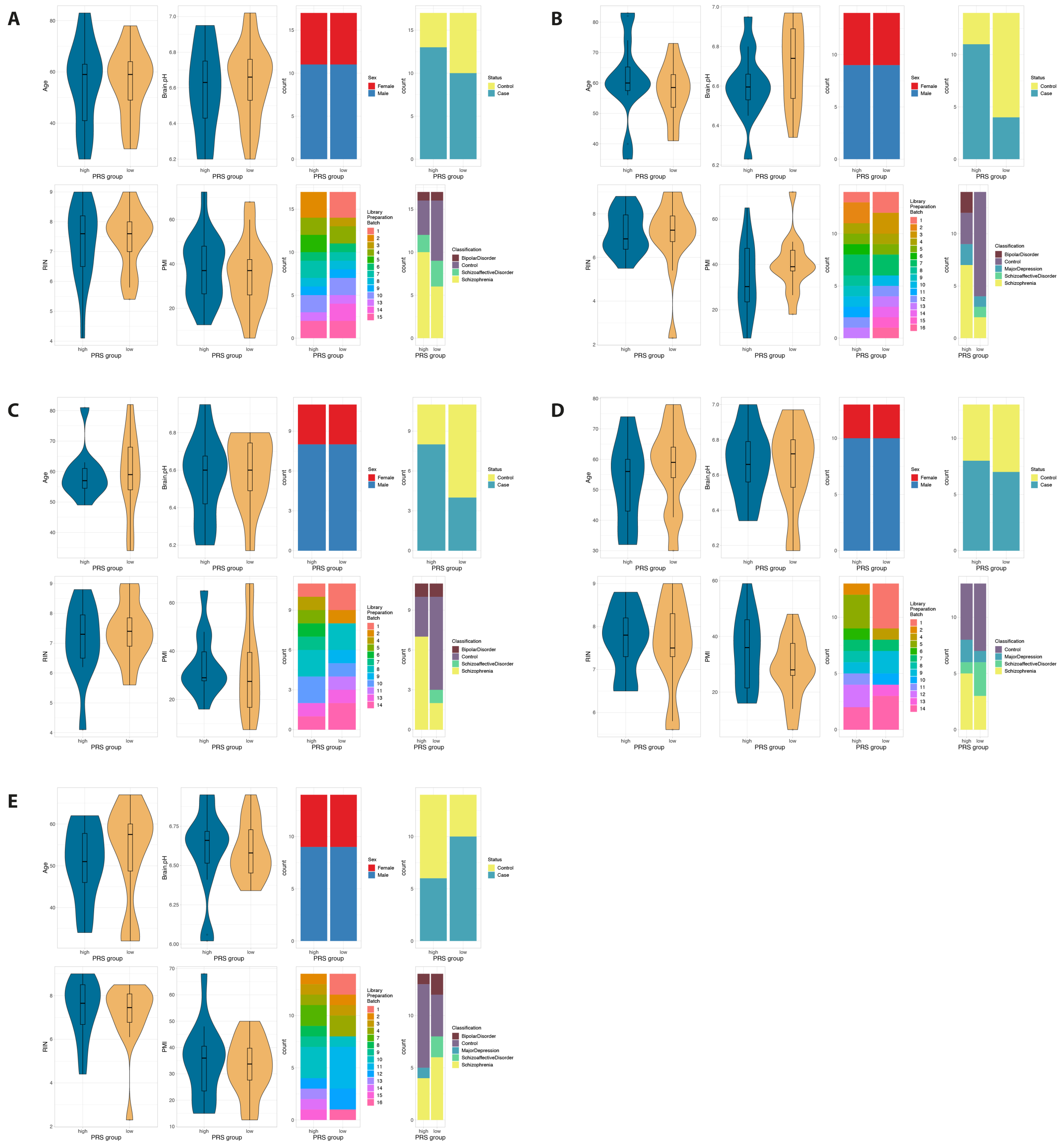

Figure S7. Definition of extreme groups for genetic risk. (A-E) Matched covariates and distribution of disease status and diagnoses for high and low risk groups for cross-disorder phenotype (A), bipolar disorder (B), major depressive disorder (C), schizophrenia (D) and height (E).

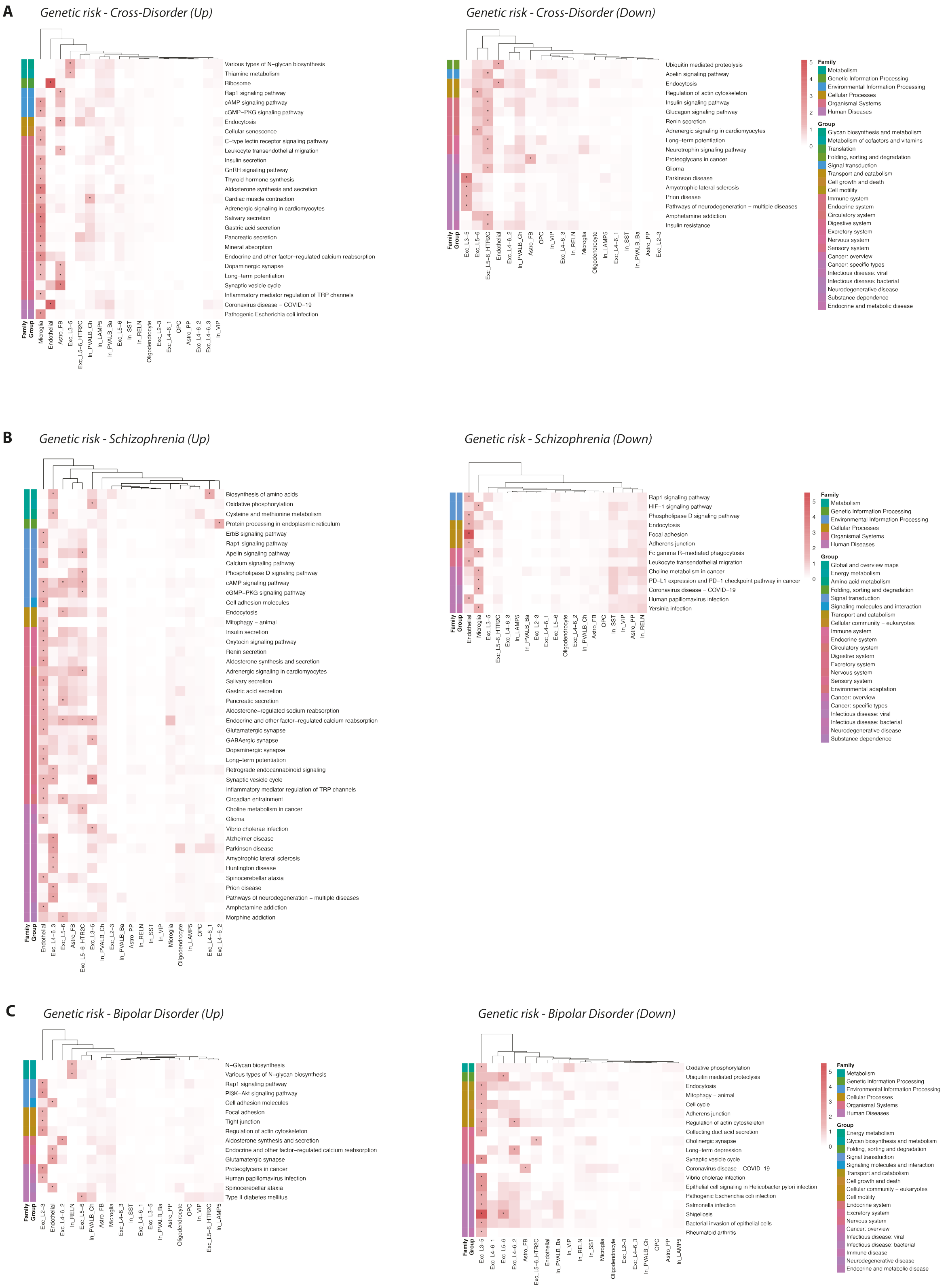
A
Genetic risk - Cross-Disorder (Up)
Various types of N−glycan biosynthesis
Thiamine metabolism
Ribosome
Rap1 signaling pathway
cAMP signaling pathway
cGMP−PKG signaling pathway
Endocytosis
Cellular senescence
C−type lectin receptor signaling pathway
Leukocyte transendothelial migration
Insulin secretion
GnRH signaling pathway
Thyroid hormone synthesis
Aldosterone synthesis and secretion
Cardiac muscle contraction
Adrenergic signaling in cardiomyocytes
Salivary secretion
Gastric acid secretion
Pancreatic secretion
Mineral absorption
Endocrine and other factor−regulated calcium reabsorption
Dopaminergic synapse
Long−term potentiation
Synaptic vesicle cycle
Inflammatory mediator regulation of TRP channels
Coronavirus disease − COVID−19
Pathogenic Escherichia coli infection
Family
Group
Microglia
Endothelial
Astro_FB
Exc_L3−5
Exc_L5−6_HTR2C
In_PVALB_Ch
In_LAMP5
In_PVALB_Ba
Exc_L5−6
In_SST
In_RELN
Oligodendrocyte
Exc_L2−3
Exc_L4−6_1
OPC
Astro_PP
Exc_L4−6_2
Exc_L4−6_3
In_VIP
Genetic risk - Cross-Disorder (Down)
Ubiquitin mediated proteolysis
Apelin signaling pathway
Endocytosis
Regulation of actin cytoskeleton
Insulin signaling pathway
Glucagon signaling pathway
Renin secretion
Adrenergic signaling in cardiomyocytes
Long−term potentiation
Neurotrophin signaling pathway
Proteoglycans in cancer
Glioma
Parkinson disease
Amyotrophic lateral sclerosis
Prion disease
Pathways of neurodegeneration − multiple diseases
Amphetamine addiction
Insulin resistance
Family
Group
Exc_L3−5
Exc_L5−6
Exc_L5−6_HTR2C
Endothelial
Exc_L4−6_2
In_PVALB_Ch
Astro_FB
OPC
In_VIP
Exc_L4−6_3
In_RELN
Microglia
Oligodendrocyte
In_LAMP5
Exc_L4−6_1
In_SST
In_PVALB_Ba
Astro_PP
Exc_L2−3
5
4
3
2
1
0
Family
Metabolism
Genetic Information Processing
Environmental Information Processing
Cellular Processes
Organismal Systems
Human Diseases
Group
Glycan biosynthesis and metabolism
Metabolism of cofactors and vitamins
Translation
Folding, sorting and degradation
Signal transduction
Transport and catabolism
Cell growth and death
Cell motility
Immune system
Endocrine system
Circulatory system
Digestive system
Excretory system
Nervous system
Sensory system
Cancer: overview
Cancer: specific types
Infectious disease: viral
Infectious disease: bacterial
Neurodegenerative disease
Substance dependence
Endocrine and metabolic disease
B
Genetic risk - Schizophrenia (Up)
Biosynthesis of amino acids
Oxidative phosphorylation
Cysteine and methionine metabolism
Protein processing in endoplasmic reticulum
ErbB signaling pathway
Rap1 signaling pathway
Apelin signaling pathway
Calcium signaling pathway
Phospholipase D signaling pathway
cAMP signaling pathway
cGMP−PKG signaling pathway
Cell adhesion molecules
Endocytosis
Mitophagy − animal
Insulin secretion
Oxytocin signaling pathway
Renin secretion
Aldosterone synthesis and secretion
Adrenergic signaling in cardiomyocytes
Salivary secretion
Gastric acid secretion
Pancreatic secretion
Aldosterone−regulated sodium reabsorption
Endocrine and other factor−regulated calcium reabsorption
Glutamatergic synapse
GABAergic synapse
Dopaminergic synapse
Long−term potentiation
Retrograde endocannabinoid signaling
Synaptic vesicle cycle
Inflammatory mediator regulation of TRP channels
Circadian entrainment
Choline metabolism in cancer
Glioma
Vibrio cholerae infection
Alzheimer disease
Parkinson disease
Amyotrophic lateral sclerosis
Huntington disease
Spinocerebellar ataxia
Prion disease
Pathways of neurodegeneration − multiple diseases
Amphetamine addiction
Morphine addiction
Family
Group
Endothelial
Exc_L4−6_3
Exc_L5−6
Astro_FB
Exc_L5−6_HTR2C
Exc_L3−5
In_PVALB_Ch
Exc_L2−3
In_PVALB_Ba
Astro_PP
In_RELN
In_SST
In_VIP
Microglia
Oligodendrocyte
In_LAMP5
OPC
Exc_L4−6_1
Exc_L4−6_2
Genetic risk - Schizophrenia (Down)
Rap1 signaling pathway
HIF−1 signaling pathway
Phospholipase D signaling pathway
Endocytosis
Focal adhesion
Adherens junction
Fc gamma R−mediated phagocytosis
Leukocyte transendothelial migration
Choline metabolism in cancer
PD−L1 expression and PD−1 checkpoint pathway in cancer
Coronavirus disease − COVID−19
Human papillomavirus infection
Yersinia infection
Family
Group
Endothelial
Microglia
Exc_L3−5
Exc_L5−6_HTR2C
Exc_L4−6_3
In_LAMP5
In_PVALB_Ba
Exc_L2−3
Exc_L4−6_1
Exc_L5−6
Oligodendrocyte
Exc_L4−6_2
In_PVALB_Ch
Astro_FB
OPC
In_SST
In_VIP
Astro_PP
In_RELN
5
4
3
2
1
0
Family
Metabolism
Genetic Information Processing
Environmental Information Processing
Cellular Processes
Organismal Systems
Human Diseases
Group
Global and overview maps
Energy metabolism
Amino acid metabolism
Folding, sorting and degradation
Signal transduction
Signaling molecules and interaction
Transport and catabolism
Cellular community − eukaryotes
Immune system
Endocrine system
Circulatory system
Digestive system
Excretory system
Nervous system
Sensory system
Environmental adaptation
Cancer: overview
Cancer: specific types
Infectious disease: viral
Infectious disease: bacterial
Neurodegenerative disease
Substance dependence
C
Genetic risk - Bipolar Disorder (Up)
N−Glycan biosynthesis
Various types of N−glycan biosynthesis
Rap1 signaling pathway
PI3K−Akt signaling pathway
Cell adhesion molecules
Focal adhesion
Tight junction
Regulation of actin cytoskeleton
Aldosterone synthesis and secretion
Endocrine and other factor−regulated calcium reabsorption
Glutamatergic synapse
Proteoglycans in cancer
Human papillomavirus infection
Spinocerebellar ataxia
Type II diabetes mellitus
Family
Group
Exc_L2−3
Endothelial
Exc_L4−6_2
In_RELN
Exc_L5−6
In_PVALB_Ch
Astro_FB
Microglia
Exc_L4−6_3
In_SST
Exc_L4−6_1
Exc_L3−5
In_PVALB_Ba
Astro_PP
Oligodendrocyte
OPC
In_VIP
Exc_L5−6_HTR2C
In_LAMP5
Genetic risk - Bipolar Disorder (Down)
Oxidative phosphorylation
Ubiquitin mediated proteolysis
Endocytosis
Mitophagy − animal
Cell cycle
Adherens junction
Regulation of actin cytoskeleton
Collecting duct acid secretion
Cholinergic synapse
Long−term depression
Synaptic vesicle cycle
Coronavirus disease − COVID−19
Vibrio cholerae infection
Epithelial cell signaling in Helicobacter pylori infection
Pathogenic Escherichia coli infection
Salmonella infection
Shigellosis
Bacterial invasion of epithelial cells
Rheumatoid arthritis
Family
Group
Exc_L3−5
Exc_L4−6_1
Exc_L5−6
Exc_L4−6_2
Astro_FB
Exc_L5−6_HTR2C
Endothelial
In_PVALB_Ba
In_VIP
In_RELN
In_SST
Microglia
Oligodendrocyte
Exc_L2−3
Exc_L4−6_3
In_PVALB_Ch
OPC
Astro_PP
In_LAMP5
5
4
3
2
1
0
Family
Metabolism
Genetic Information Processing
Environmental Information Processing
Cellular Processes
Organismal Systems
Human Diseases
Group
Energy metabolism
Glycan biosynthesis and metabolism
Folding, sorting and degradation
Signal transduction
Signaling molecules and interaction
Transport and catabolism
Cell growth and death
Cellular community − eukaryotes
Cell motility
Endocrine system
Excretory system
Nervous system
Cancer: overview
Infectious disease: viral
Infectious disease: bacterial
Immune disease
Neurodegenerative disease
Endocrine and metabolic disease

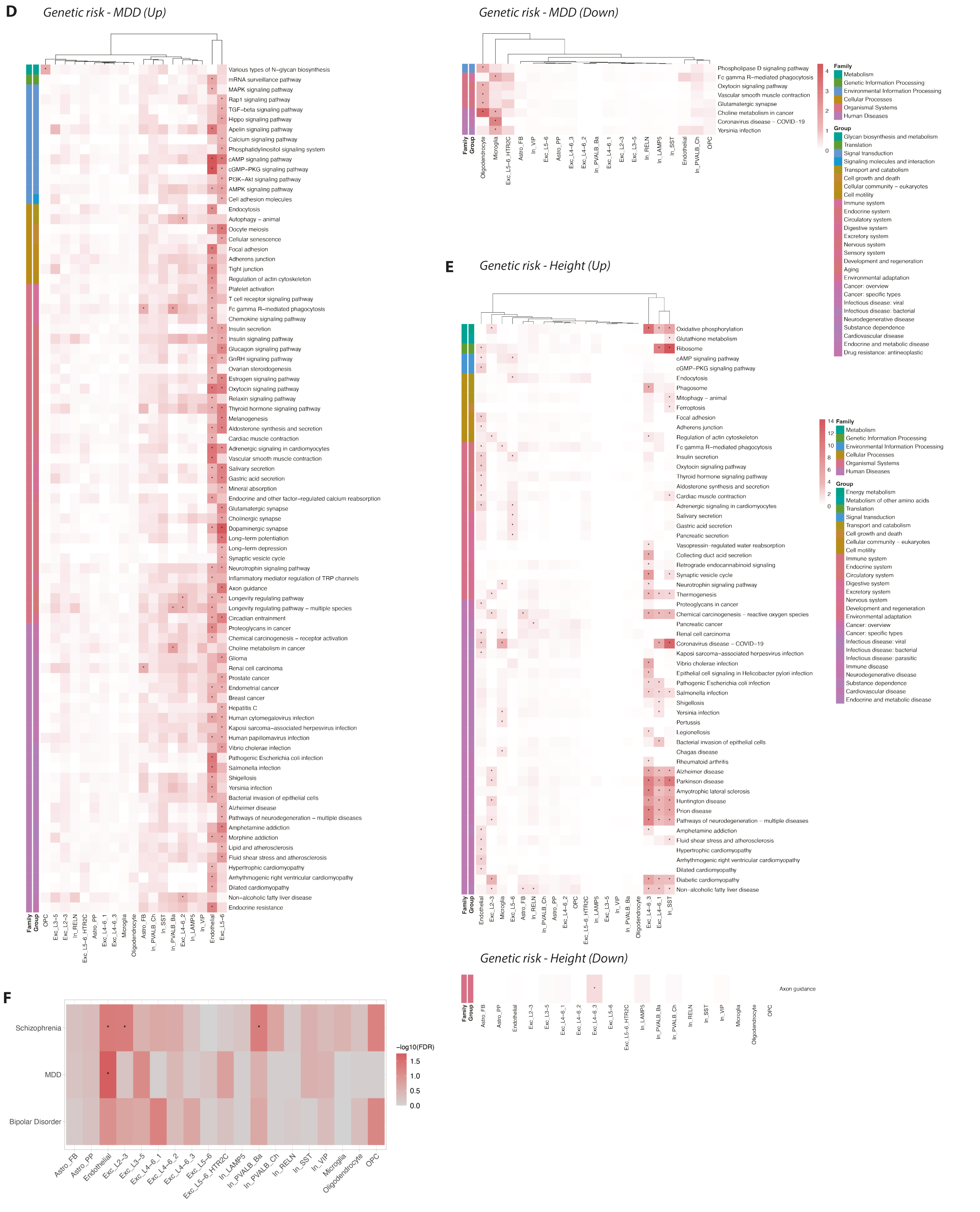

Figure S8. Dysregulations in gene expression between extreme genetic risk groups.(A-E) Results of KEGG pathway enrichment analysis for 250 most up- and downregulated genes per cell type between extreme genetic risk groups for cross-disorder phenotype (A), schizophrenia (B), bipolar disorder (C), MDD (D), and height (E). Left heatmap of each panel shows enrichment results for upregulated genes, while right heatmap of each panel shows results for downregulated genes. All pathways significantly enriched in at least one cell type are included into the heatmap. Color represents -log$_{10}$-transformed FDR values and asterisks indicate significance (FDR $\leq$ 0.05). Colored

annotations of the pathways on the left side of each plot indicate to which pathway group and family a pathway belongs. The dendrograms visualize k-means clustering of cell types according to enrichment results. (F) Results of GWAS enrichment analysis in schizophrenia DE risk genes using H-MAGMA (*36*) for GWAS hits of bipolar disorder, major depressive disorder and schizophrenia. Color indicates -$\log_{10}$-transformed FDR values and asterisks indicate significance (FDR ≤ 0.05).

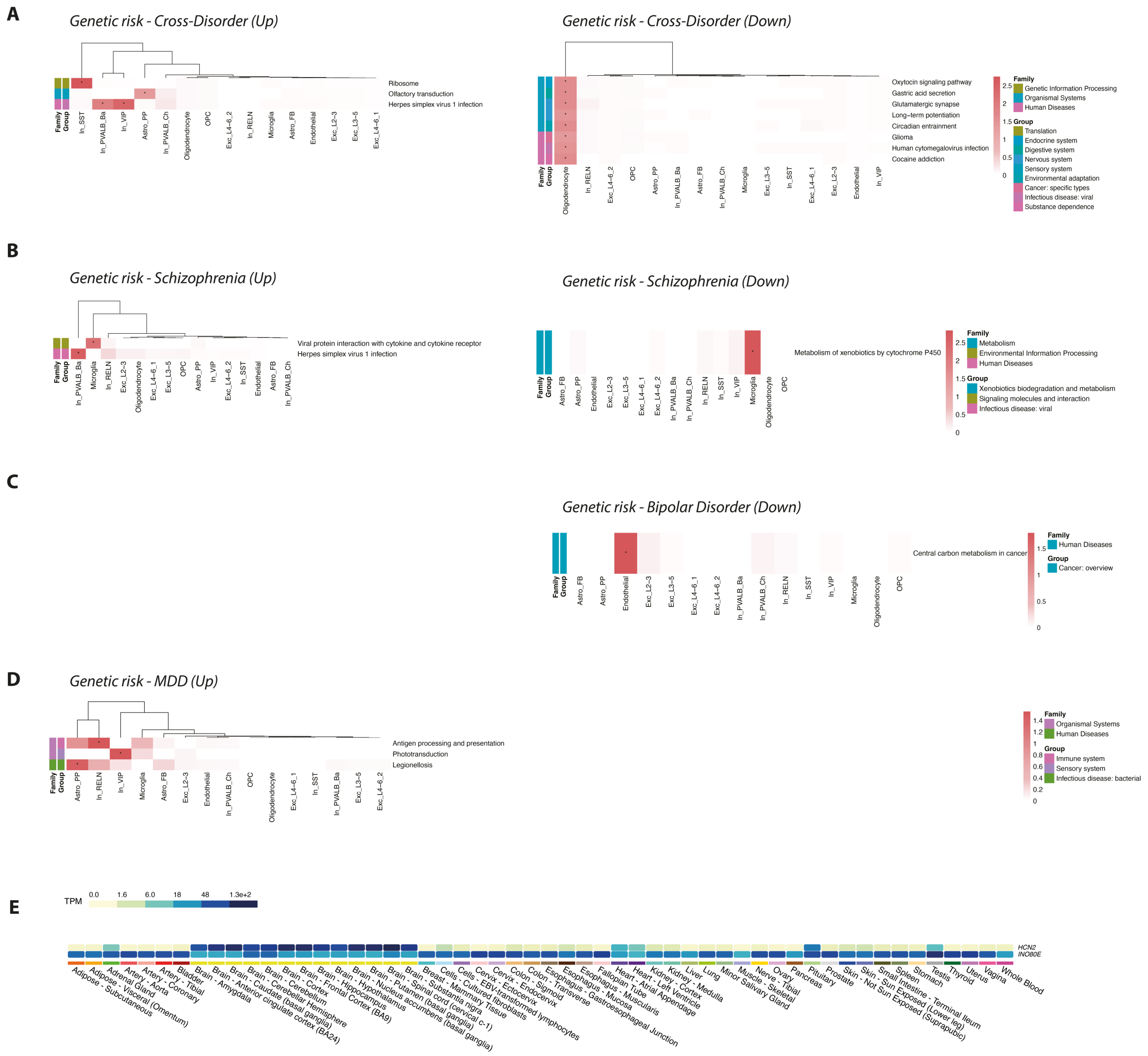

Figure S9. Dysregulations in chromatin accessibility between extreme genetic risk groups. (A-D) Results of KEGG pathway enrichment analysis for 250 most up- and downregulated genes per cell type between extreme genetic risk groups for cross-disorder phenotype (A), schizophrenia (B), bipolar disorder (C), and MDD (D). No pathways were enriched for height DA risk genes. Left heatmap of each panel shows enrichment results for upregulated genes, while right heatmap of each panel shows results for downregulated genes. All pathways significantly enriched in at least one cell type are included into the heatmap. Color represents -$\log_{10}$-transformed FDR values and asterisks indicate significance (FDR ≤ 0.05). Colored annotations of the pathways on the left side of each plot indicate to which pathway group and family a pathway belongs. The dendrograms visualize k-means clustering of cell types according to enrichment results. (E) Gene expression levels (i.e. transcript per million, TPM) for *HCN2* and *INO80E* across multiple human tissues, as sourced from the GTEx portal (*33*).

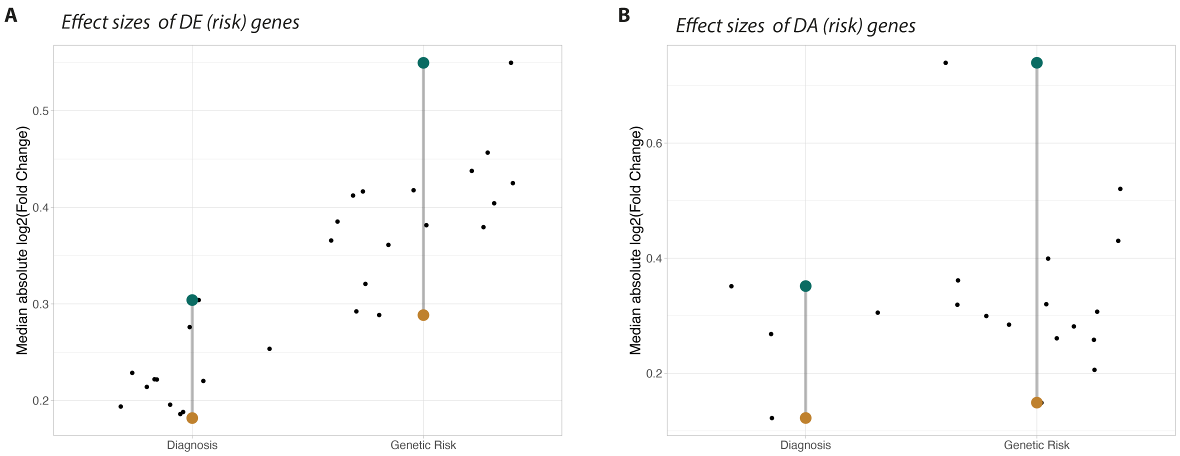

Figure S10. Range of effect sizes for clinical diagnosis and genetic risk. (A-B) Visualizations of the range of absolute median log_2-transformed fold changes per cell type for DE (risk) genes (A) and DA (risk) genes (B). The vertical lines represent the range of effect sizes across cell types with the colored dots representing the minimum and maximum effect size each. Small black dots represent the median effect sizes for specific cell types.

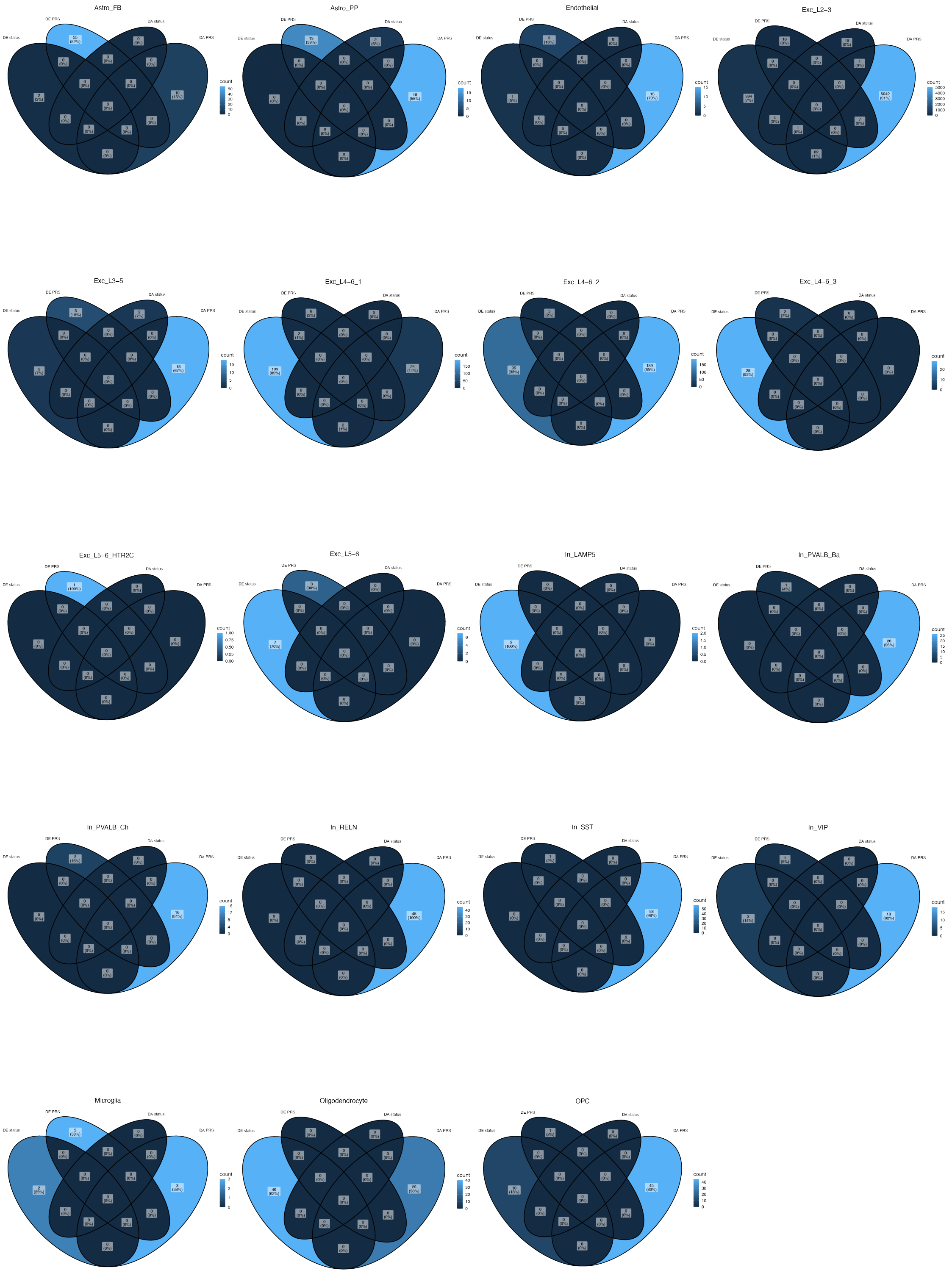

Figure S11. Differentially expressed and accessible genes between cases and controls and genetic risk groups. Venn diagram for each cell type comparing DE and DA genes between disease status with DE and DA risk genes between genetic risk groups. DE and DA risk genes are aggregated across the cross-disorder, schizophrenia, MDD and bipolar disorder GWAS studies.

Table S1-S15 in separate file

Table S1. NSW Brain Cohort Overview.

Table S2. NSW Brain Cohort Covariates and Inclusion in Downstream Analyses.

Table S3. Differences in Cell Type Proportions between snRNA-seq and snATAC-seq data.

Table S4. Polygenic risk scores (PRS) for NSW Brain Cohort.

Table S5. Number of cases and diagnoses in high and low risk groups.

Table S6. Number of Samples after Outlier Removal.

Table S7. Differentially expressed genes between cases and controls.

Table S8. Differentially accessible genes between cases and controls.

Table S9. Differentially expressed and accessible genes between cases and controls.

Table S10. Differentially expressed genes between extreme genetic risk groups.

Table S11. Differentially accessible genes between extreme genetic risk groups.

Table S12. Transcription factor motif enrichments for *INO80E* and *HCN2*.

Table S13. Differentially expressed genes between cases and controls using nuclei count as a covariate.

Table S14. Differentially expressed genes for schizophrenia subsample.

Table S15. Differentially expressed genes for stratified cross-disorder subsample.
